# An Innovative PTD-IVT-mRNA Delivery Platform for CAR Immunotherapy of ErbB(+) Solid Tumor Neoplastic Cells

**DOI:** 10.3390/biomedicines10112885

**Published:** 2022-11-10

**Authors:** Sofia K. Georgiou-Siafis, Androulla N. Miliotou, Charikleia Ntenti, Ioannis S. Pappas, Lefkothea C. Papadopoulou

**Affiliations:** 1 Laboratory of Pharmacology, School of Pharmacy, Faculty of Health Sciences, Aristotle University of Thessaloniki, 54124 Thessaloniki, Macedonia, Greece; 2 Laboratory of Pharmacology and Toxicology, Faculty of Veterinary Medicine, University of Thessaly, 43100 Karditsa, Thessaly, Greece; 3 Department of Health Sciences, KES College, Nicosia 1055, Cyprus; 4 1st Laboratory of Pharmacology, School of Medicine, Faculty of Health Sciences, Aristotle University of Thessaloniki, 54124 Thessaloniki, Macedonia, Greece

**Keywords:** chimeric antigen receptor (CAR) immunotherapy, IVT-mRNAs, protein transduction domain (PTD), PTD-IVT-mRNAs, delivery, NK-92 cells, T1E scFV, ErbB, HSC-3 cells, MCF-7 cells

## Abstract

Chimeric antigen receptor (CAR) immunotherapy includes the genetic modification of immune cells to carry such a receptor and, thus, recognize cancer cell surface antigens. Viral transfection is currently the preferred method, but it carries the risk of *off*-target mutagenicity. Other transfection platforms have thus been proposed, such the in vitro transcribed (IVT)-mRNAs. In this study, we exploited our innovative, patented delivery platform to produce protein transduction domain (PTD)-IVT-mRNAs for the expression of *CAR* on NK-92 cells. CAR T1E-engineered NK-92 cells, harboring the sequence of T1E single-chain fragment variant (scFv) to recognize the ErbB receptor, bearing either CD28 or 4-1BB as co-stimulatory signaling domains, were prepared and assessed for their effectiveness in two different ErbB(+) cancer cell lines. Our results showed that the PTD-IVT-mRNA of *CAR* was safely transduced and expressed into NK-92 cells. CAR T1E-engineered NK-92 cells provoked high levels of cell death (25–33%) as effector cells against both HSC-3 (oral squamous carcinoma) and MCF-7 (breast metastatic adenocarcinoma) human cells in the co-incubation assays. In conclusion, the application of our novel PTD-IVT-mRNA delivery platform to NK-92 cells gave promising results towards future CAR immunotherapy approaches.

## 1. Introduction

The immune system has the vital ability to detect tumor cells and respond to eliminate them. However, neoplastic cells develop mechanisms to finally escape from immune surveillance [1]. Furthermore, tumor-associated genetic alterations promote the existing heterogeneity among different types of cancer and individuals, and hence there is an urgent need for personalized cancer therapies. 

CAR therapy is a personalized therapeutic approach, including the ex vivo genetic modification of autologous immune cells to selectively target cancer cells [2]. The harvested immune cells, mainly T cells and natural killer (NK) cells, are genetically modified with the coding sequence (CDS) of the CAR, usually mediated by viral transfection. Next, the genetically modified T cells are ex vivo cultured for further expansion. Prior to the subsequent re-infusion of CAR T cells into patients, lymphodepleting chemotherapy is required to allow the infused CAR T cells to remain in circulation for a longer period and/or to reduce the anti-CAR immune response [3]. 

CARs recognize cell surface, tumor-associated antigens (TAAs), via the single-chain variable fragment (scFv), located extracellularly in the modified immune cell [4]. The extracellular part of CAR is linked to intracellular signaling molecules, the CD3ζ chain and CD28 or 4-1BB co-stimulatory domains (second generation CARs), while third generation CARs combine more than one co-stimulatory molecule. Pro-inflammatory cytokines can also be incorporated in order to enhance the innate immune response. The use of different co-signaling domains can contribute to alterations in the metabolic profile of the respective CAR cells [5].

Clinical trials have shown spectacular efficacy in patients with end-stage ALL using anti-CD19 CAR T cells with up to 93% complete remission [6]. Back in 2017, chimeric antigen receptor (CAR) immunotherapy revolutionized the cancer treatment strategies with the first-in-modality FDA approval of Tisagenlecleucel for patients with refractory or in relapse B-cell acute lymphoblastic leukemia (B-ALL). The FDA authorized five more CAR T cell therapies, which are available for pediatric/young adult patients for use only in hematological malignancies [7]. 

In addition, high-edge CAR immunotherapy is being developed for solid tumors as well, with clinical studies being performed with excellent remission results in ovarian [8], breast [9], prostate [10], brain [11], renal [12], gastric [13], pancreatic [14], lung [15], liver [16] and colorectal cancer [17]. However, CAR immunotherapy for solid tumors faces fundamental barriers to its effectiveness, which is strongly dependent on the tumor type, the disease stage and the corresponding genetic profile. Diverse expression of TAAs among cancer cells promote heterogeneity regarding CAR cell efficiency. Moreover, a limited access of CAR cells into the tumor tissue and CAR cell exhaustion have been recorded [18]. However, the most important challenge that CAR cells face with solid tumors is the immunosuppressive tumor microenvironment [19]. Τhere are also several challenges that escort CAR therapeutic strategies into the clinic. These limitations are related to patient enrollment into clinical trials and inclusion criteria, as well as the increased cost.

Regarding adverse reactions, CAR therapies have been associated with the cytokine release syndrome (CRS), a systemic response of excess inflammation, through cytokine release (IL-1, IL-6, IL-8, IFN-γ, GM-CSF) that can cause multiple organ failure and even death [20]. In addition, neurotoxicity [21] as well as on-target/off-tumor, on-target/on-tumor, off-target/off-tumor [3], graft-versus-host disease (GvHD) [22] and CAR T cell-associated hemophagocyticlymphohistiocytosis (HLH)/macrophage activation syndrome (MAS) have been recorded following CAR T cell infusion [23].

Since CAR immunotherapy is an extremely costly and elaborate approach, the accessibility to this therapy remains limited [24]. Furthermore, the laborious leukapheresis needed to harvest autologous and healthy T cells that is associated with poor quality and limited quantity comes to add to the obstacles of the wide application of this therapy. Thus, off-the-shelf products are in the spotlight, promoting standardization and developing programmable systems of CAR therapies, named universal CAR therapies [25]. Manufacturing ready-to-use batches of such *CAR* cells will facilitate rapid treatment (preventing disease progression) and less cost for the patient. Since NK cells also possess a remarkable cytotoxic potential (through their involvement in natural and acquired immunity), they can be considered as candidate cells, in addition to T cells, in CAR therapy [26]. NK-92, a homogenous, stable, human cell population serves commonly as a cell line model for NK cells [27,28]. Thus, keeping in mind all these considerations, the NK-92 cell line is an attractive platform for off-the-shelf CAR therapy since it also avoids the graft/host reaction. In fact, several clinical studies have shown the effectiveness of engineered NK-92 cells against a variety of cancers, including blood malignancies and solid tumors [29,30].

Currently, viral transfection, using retroviruses, adenoviruses and adeno-associated viruses is the preferred procedure to transduce the CDS of *CAR* into the immune cells. Viral vectors stably integrate the genetic material into the host genome. There is a considerable risk because of random integration/mutagenesis into the genome sites. Immune-mediated toxicity, caused by long-term persistence and activity of engineered T cells, has been recorded as an additional risk [31]. There are also restrictions on the size and number of genes that can be packed into these vectors. In addition, heterogeneous copy numbers can result in T cell populations with highly variable cytotoxic capabilities due to different expression levels of the *CAR* [3]. In vivo, and not ex vivo, *CAR* delivery has also been proposed; however, safety (short- and long-term) and efficacy remain challenging [24].

Other ex vivo transfection platforms have been proposed, including the IVT-mRNAs (in vitro transcribed-mRNAs). IVT-mRNA technology is an innovative technology in the biopharmaceutical industry, associated with potential for advanced genetic therapies. IVT-mRNA is a synthetic mRNA which facilitates the transient expression of a gene of interest, exploiting the cell’s translational machinery. IVT-mRNA is considered a safe and dynamic alternative compared to most viral DNA/RNA vectors for use in clinical applications, mainly due to its biological origin, its transient nature and the lack of host genome interference [32]. Over the years, a number of structural and chemical modifications have been proposed to improve the IVT-mRNAs stability and avoid the immunological response mechanisms [32,33]. A common structural modification of the IVT-mRNA is the addition of anti-reverse cap analogs (ARCAs), increasing mRNA stability and translation rates [32,34]. Optimizing the IVT-mRNA can also be achieved by the incorporation of modified nucleosides, such as 5mC (5-methylcytidine) and Ψ (pseudo-uridine) [35,36]. Polyadenylation as well as the addition of murine 5′-UTR and human 3′-UTR of the *α*-*globin* and *β-globin* mRNAs offer great advantages regarding IVT-mRNA stability [32,37]. 

However, the intracellular transduction of IVT-mRNAs remains a challenge. Cell membrane disruption methods are an option but may lead to quite high levels of cell death percentages as well heterogeneity in transfection efficiency [38]. The most preferable methods, already contributed in SARS-CoV-2 mRNA vaccines, consist of lipid-based systems, such as the lipid-like nanoparticles (LNPs) [39,40]. 

In this study, we present the application of the protein transduction domain (PTD) technology for the delivery of IVT-mRNAs of *CAR*. PTDs are small peptides in length (less than 30 aa), having the ability to transduce biological barriers and carrying intracellularly a variety of different sized cargoes [41]. We have already provided experimental evidence for the successful transduction of recombinant proteins, in the context of protein replacement therapy (PRT) for monogenic/metabolic disorders [42,43,44,45,46]. Furthermore, our research group designed and developed a novel PTD-IVT-mRNA delivery platform through a patented covalent chemical reaction. This novel delivery platform showed promising results in an in vitro study, as a potential PRT approach of a mitochondrial disorder, due to *SCO2* deficiency as well as of *β*-thalassemia [42]. 

To further develop this novel delivery platform, our research group proceeded to a thorough study of CAR immunotherapy, targeting oral squamous cell carcinoma (OSCC), a highly resistant and solid tumor of poor prognosis overexpressing ErbB receptors. The CAR T1E-NK-92 cells were genetically engineered via the transduction of the PTD-IVT-mRNAs of the CAR sequences needed for the recognition of the ErbB receptor. CAR engineered cells are designed to express either CD28 or 4-1BB co-stimulatory signaling domain to study their cytotoxic effectiveness. Finally, CAR T1E-engineered NK-92 cells are assessed through co-incubation assays with OSCC and MCF-7 (breast metastatic adenocarcinoma cell lines) to investigate their cytotoxic potential as an effective CAR immunotherapy approach to ErbB+ cancers.

## 2. Materials and Methods

### 2.1. Cell Culture and Reagents 

ΝΚ-92 cells (ATCC, CRL-2407) are an IL-2-dependent natural killer cell line, isolated from a 50-year-old male with non-Hodgkin’s lymphoma. This cell line was used as effector cells (E) and it was a kind gift of Professor Lorenzo Moretta (Immunology Research Area, IRCSS Bambino Gesù Pediatric Hospital, Rome, Italy). NK-92 cells were cultured as described previously [47]. Interleukin-2 (CST, Danvers, MA, USA) (at 50 ng/mL), L-glutamine (Biosera, Rue de la Caille, France) (at 2 mM) and gentamicin (Applichem GmbH, Darmstadt, Germany) (at 50 μg/mL) were added in the NK-92 cells medium at the indicated concentrations. Both HSC-3 cells (Sigma-Aldrich, St Luis, MO, USA, SCC193) and MCF-7 cells (ATCC, HTB-22) (kindly donated by Professor Ioannis Vizirianakis, School of Pharmacy, A.U.Th) were cultured in DMEM (Gibco, Thermo Fisher Scientific, Waltham, MA, USA) supplemented with 10% fetal bovine serum (FBS, Gibco). The previously established K562 human erythroleukemia cells (CCL-243^TM^) [48] were routinely cultured in our laboratory of Pharmacology with RPMI-1640 medium (Gibco) in presence of FBS (10%), as previously shown [45,46,49]. A mixture of penicillin/streptomycin/antimycotics (Gibco) was regularly added at all cultures, maintained at 37 °C in 5% CO_2_ atmosphere. The cell lines used are of human origin.

### 2.2. Design, Construction and Cloning of CAR T1E28z and CAR T1E4-1BBz Sequences

**Figure 1:**
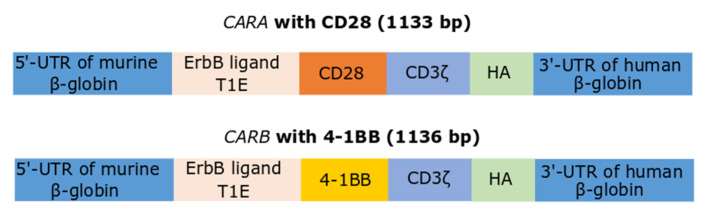
Schematic illustration of the two IVT-mRNAs employed in the present study that correspond to the second generation *CARA* and *CARB*. Both constructs harbored the T1E scFv as well the CD3ζ intracellular T cell signaling sequence and fused either to CD28 (*CARA*) or 4-1BB (*CARB*) co-stimulatory endodomains.

The sequence for T1E single-chain fragment variant (scFv) was kindly offered by Professor John Maher (King’s College London, King’s Health Partners Integrated Cancer Center, Department of Research Oncology, Guy’s Hospital Campus, London, UK) [50]. This sequence for T1E was synthesized by order (Eurofins Genomics, Louisville, KY, USA). Several other sequences (illustrated at Figure 1) prior to and after the scFv sequence were included during synthesis. In particular, the scFv sequence was preceded by the 5′-untranslated region (UTR) of murine *β-globin*, ensuring proper translation in eukaryotic cells. The T1E scFv was followed by the co-stimulatory endodomains (CD28 or 41BB), as well by the intracellular T cell signaling sequence, CD3ζ and the 3′-UTR of human *β-globin*. The UTRs of *β-globins* were used, as the mRNAs of *globins* are characterized by long half-lives. In this way, two different constructs for second generation CAR were yielded, *CAR* T1E28z and *CAR* T1E4-1BBz, bearing either CD28 or 41BB, and named for our convenience *CARA* and *CARB*, respectively. The epitope for hemagglutinin (HA) was inserted to probable auxiliary facilitate the detection of CAR constructs in subsequent experiments. Both *CAR* sequences were TA-cloned into pGEM-T easy vector (Promega, Madison, WI, USA), an appropriate vector for in vitro transcription (Figure S1A,B). Proper sense orientation of cloning was checked by restriction enzyme digestions (Figure S1C,D) and verified by sequencing (CEMIA, Larissa, Greece). 

### 2.3. Production of IVT mRNAs

The pGEM plasmids, either with the *CARA* or the *CARB* insert, after linearization with NdeI (NEB, Hitchin, UK), were phenol chloroform-extracted, serving thus as the templates for the in vitro transcription. T7 RNA polymerase-promoted transcription conducted in the presence of anti-reverse cap analogs [Hiscribe^TM^ kit mRNA synthesis (NEB)] was associated with efficient translation rates [51]. After completion of the translation, DNase I and poly-A polymerase were added, followed by LiCl precipitation. Agarose gel electrophoresis of the resulting IVT-mRNAs, prior heat-denatured (70 °C, 10 min) in RNA formamide-loading dye (90% formamide, 10 mM EDTA, 0.25% xylene cyanol FF, and 0.25% bromophenol blue) ensured proper IVT-mRNAs production, as well poly-adenylation processes (Figure S2). The quantity of IVT-mRNAs was assessed via a UV spectrophotometer (Nanodrop; Thermo Fisher Scientific, USA). RNA (RiboRuler LR) and DNA (FastGene) ladders were products of Thermo-Scientific and Nippon Genetics Europe GmbH (Düren, Germany). 

### 2.4. Conjugation of the Selected PTD to the IVT-mRNAs of CARA and CARB

The IVT-mRNAs were covalently conjugated to the PTD via our novel IVT-mRNA delivery platform, entitled «Method for the development of a delivery platform to produce deliverable PTD-IVT-mRNA therapeutics» (Greek patent, No.: 1010063) [42]. The selected PTD (PFVYLI) (ordered by Genecust, Boynes, France) is conjugated via amide bond to puromycin (Applichem), serving as the linker for the conjugation of the PTD to the IVT-mRNA. Reagents employed in the conjugation reaction of PTD to the IVT-mRNAs were: T7 RNA ligase and the RNaseOut^TM^ recombinant ribonuclease inhibitor (Invitrogen); T4 polynucleotide kinase (NEB); EDC-HCl[N-(3-Dimethylaminopropyl)-*N*′-ethylcarbodiimide hydrochloride] (Sigma-Aldrich); RNase A DNase free (used at the PTD-IVT-mRNAs stability assays) (Applichem GmbH).

### 2.5. Transfection of NK-92 Cells (Effector Cells, Ε) with the PTD-IVT-mRNAs for CARA and CARB

Exponentially grown NK-92 cells, rinsed twice with PBS buffer, were suspended in Opti-MEM I Reduced serum medium (Gibco, Thermo) at a concentration of 10^6^ cells per 0.5 mL of medium, and 0.5 μg of PTD-IVT-mRNA (as per IVT-mRNA) per 10^6^ cells was added to culture medium. After that, IL-2 was added, and 2 h later, cells were supplemented with the regular medium (at 2× of the Opti-MEM volume). Lipofectamine 2000 (Invitrogen, Waltham, MA, USA), a known experimental transfection reagent, was used according to manufacturers’ instructions. IVT-mRNA was again added at 0.5 μg/10^6^ cells. The same procedures were followed in the transfection of pro-erythroid K562 cells.

### 2.6. RNA Isolation, Reverse Transcription (RT)-PCR and Quantitative (q)PCR

Total RNA extraction from cells was performed through standard procedures [52]. For one-step RT-PCR (KAPA SYBR Fast one step, Kapa Biosystems Ltd., Basel, Switzerland), 1 μg of total RNA was used per reaction. The reaction conditions were: 95 °C for 3 min, 35 cycles of 95 °C for 5 s, 65 °C for 20 s and 72 °C for 1 min, followed by incubation at 72 °C for 3 min. For the qPCR analysis, 1.5 μg of total RNA served as the template for first-strand cDNA synthesis (Takara Bio, San Jose, CA, USA), using oligo-dT. 5 μL of each cDNA (diluted at 1:20) served thereafter as the template for subsequent qPCR, employing KapaSYBR (Kapa Biosystems Ltd.) at Applied Biosystems 7500 Fast Real-Time PCR System. The primers (Eurofins Genomics, Ebersberg, Germany) used are: *β-ACTIN* forward: 5′-AGAGCTACGAGCTGCCTGAC-3′ and reverse: 5′-AGC ACT GTG TTG GCG TAC AG-3′, *CAR* (short fragment, 272 bp) forward: 5′-TCGCACGATGGATACTGCCT-3′ and reverse: 5′AGCACCCAAAAGGGCTTAGA-3′, *CAR* (long fragment, 1080 bp) forward: 5′- GGAAACAAAGCAATCTATTCTG-3′ and reverse: 5′- AGG CAG AAT CCA GAT GCT C-3′. The expression levels of *ACTIN*, used as an internal control, were amplified for each sample. The short fragment of *CAR* was amplified in qPCR. The reaction conditions of qPCR were: 95 °C for 3 min, 40 cycles of 95 °C for 3 s and 67 °C for 30 s, followed by incubation at 72 °C for 3 min. A single product for each qPCR reaction was verified by melting curve analysis as well as gel electrophoresis.

### 2.7. Preparation of Protein Cellular Lysates, Subcellular Fractionation and Western Blot Analysis

Cell lysis was achieved by using a lysis buffer (consisting of 50 mM Tris-HCl, pH 7.5, 150 mM NaCl, 1% SDS, and 1% Triton X-100) adjusted for the efficient extraction of membranous proteins [53]. Cell lysates were passed through a 29 g needle and after centrifugation (8000 rpm, 20 min), supernatant was collected. Subcellular fractionation of cells in membranous and cytoplasmic proteins was conducted, as described previously [54]. Protease inhibitors were purchased by Panreac-ITW Reagents (Germany). SDS-PAGE and immunoblots were carried out by standard procedures [55]. The following antibodies were used: anti-CD3ζ.IgG (sc1239), anti-β-actin.Ig.G (sc-47778) (Santa Cruz Biotechnology, Dallas, TX, USA) and anti-ErbB2.IgG (HPA001383) (Atlas Antibodies, Bromma, Sweden). Blots were developed by chemiluminescence using anti-mouse and anti-rabbit horseradish peroxidase (HRP)–conjugated antibodies (Santa Cruz Biotecnology), suitable HRP substrate (Immobilon Forte, Merck-Millipore, MA, USA) and X-ray autoradiography films (Fujifilm, Tokyo, Japan). The quantitation of protein bands was conducted in Image J (Image J, Rasband, W.S., ImageJ, U. S. National Institutes of Health, Bethesda, MD, USA) via automatic selection.

### 2.8. Assessment of Cell Growth and Viability of NK-92 Cells (Εffector Cells, Ε) 

Cell growth of NK-92 cells was determined on a CyFlow Cube 8 flow cytometer (SysmexPartec, Münster, Germany), exploiting the number of cells counted per specified volume analyzed. Cell death was assessed via probing NK-92 cells with Zombie Red, an amino-reactive fluorescent dye that is permeant to cells with compromised membranes (Zombie Red, Biolegend, San Diego, CA, USA). Cells were stained with the dye (1:200, in PBS) for 15 min at room temperature prior to the analysis in the flow cytometer. Analysis of the data took place using FlowJo software (BD Biosciences). Doxorubicin (Adriblastina)-treated NK-92 cells were also analyzed in parallel.

### 2.9. Co-Incubation Experiments of NK-92 Cells (Effector Cells, E) with HSC-3 or MCF-7 Cells (Target Cells, T)

NK-92 cells were transfected (Section 2.5) with the PTD-IVT-mRNAs for 36 h. 2,3-butanediol (Fluorechem, Glossop, UK) [56] was added at 10 μM. At the end of the transfection period, NK-92 cells were washed out from the culture medium containing the PTD-IVT-mRNAs, rinsed once with PBS and suspended in fresh medium at 3 × 10^5^ cells/sample. The co-incubation period lasted 16 h and included the addition of NK-92 cells (effector cells, E) over the HSC-3 cells or MCF-7 cells (target cells, T), priorly cultured overnight at 5 × 10^4^ cells/well, yielding a ratio of E:T cells of 10:1. To achieve the 20:1 ratio, NK-92 cells were suspended at 6 × 10^5^ cells/sample, respectively. In the same manner, a E:T ratio of 5:1 was achieved. Higher E:T ratio than 20:1 was not applied due to the observed cytotoxicity not being increased in E:T ratios above 10:1.

The following two co-incubations treatments were also included in the experimental process: HSC-3 or MCF-7 cells (T) co-incubated either with NK-92 cells left untreated or with NK-92 cells transfected with the Lipofectamine-IVT-mRNAs. These co-incubations were assessed for cytotoxicity complementary to the cytotoxicity induced by the PTD-IVT-mRNAs-engineered NK-92 cells, which was our main goal. Moreover, each subpopulation of cells (HSC-3, MCF-7 and/or NK-92 cells) was cultured separately to assess cell death percentage irrespective of the co-incubation treatment. Each experimental treatment was performed in triplicate.

Cell death was assessed in single cultures after centrifuging cells, while in the co-incubation treatments, target cells were detached by trypsinization and included in the pool of NK-92 cells. Lastly, cells were suspended in culture medium in a volume of 100 or 200 μL, for treatments of one or two sub-populations, respectively. Cell death was determined by the trypan blue exclusion assay and expressed as percentage. Firstly, cell death (attributed to mixing of the two sub-populations, without the co-incubation period) was calculated by determining the medium value of cell death between the target cells, cultured alone, and the corresponding NK-92 cells, cultured also separately. Then, the percentage of cell death induced by the co-incubation with NK-92 cells was calculated by subtracting the percentage of cell death attributed to mixing of the sub-population from the cell death percentage measured at each co-incubation experiment. By doing so, the percentage of cell death induced by the co-incubation treatment was determined. However, NK-92 cells and target cells could not be discriminated by the trypan-blue microscopy. Our percentages represent the sum of the two sub-populations.

### 2.10. Determination of Cell Death in the Co-Incubation Experiments

Cell death was assessed via the trypan blue (Sigma-Aldrich)-exclusion assay, via counting at least 400 cells in each sample. To enhance the sensitivity of the assay, cells, after the co-incubation period, were collected in fresh RPMI with NiSO_2_ (120 mM) and incubated for 5 min at room temperature [57]. Thereafter, cells were centrifuged, suspended again in the medium and assessed by optical microcopy. 

As an additional assay for cell death assessment, cells were probed sequentially in PBS with calcein violet and propidium iodide (according to Biolegend’s guides) for 30 and 15 min, respectively, at 37 °C. Then, cells were centrifuged and suspended in culture medium (a colorless version, the RPMI-11835, Gibco), allowed to recover for 30 min and proceeded to fluorescent microscopy imaging. Three different fields were scanned per sample. For each field, images were acquired (Zeiss Axio Imager) in the phase contrast, green and red filters. The intensity of each fluorescent microscopy image was quantified by automatic selection in Image J.

### 2.11. Statistical Analysis and Software

The experiments presented were carried out in three biological replicates and the mean ± SD is presented. One-way ANOVA comparison between groups was performed. Statistical significance was calculated via *t*-test analysis, assuming equal variance. The graphs were designed in Graph Pad Prism version 8.0.0 for Windows, GraphPad Software (San Diego, CA, USA), while figures were composed in InKscape (Inkscape Project. (2020)).

## 3. Results

### 3.1. Synthesis of the PTD-IVT-mRNAs of CARA and CARB

The constructs for the two second generation CAR-receptors are presented at Figure 1. Both sequences bear the T1E scFv, acting as the ErbB-receptor ligand [50], followed by the co-stimulatory domains (either CD28 or 41BB), as well by the intracellular T cell signaling sequence CD3ζ (*CARA* and *CARB* constructs, respectively). The 5′- and 3′-UTRs of *β-globin* genes were inserted towards the proper translation and stabilization of the IVT-mRNAs. The sequence of murine *β-globin* 5′-UTR was modified to include a strong Kozak sequence [42]. 

**Figure 2:**
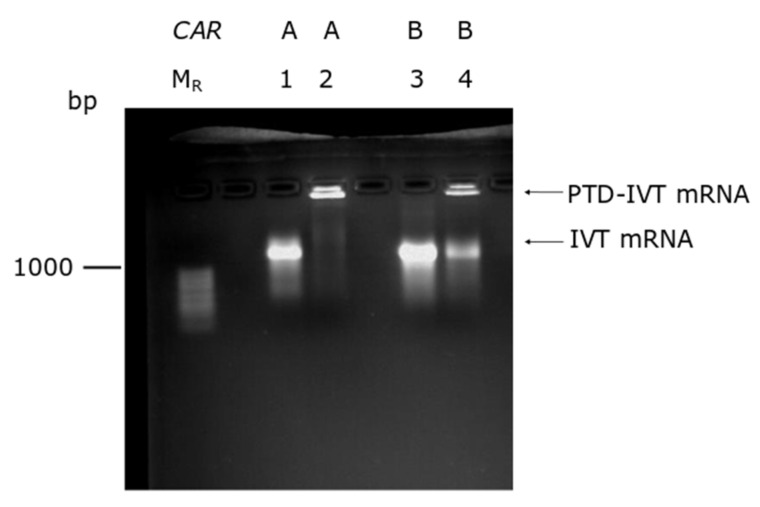
Band-shift assay of the PTD-IVT-mRNAs. Naked IVT-mRNAs and the corresponding PTD-IVT-mRNAs were heat-denatured at 70 °C for 10 min and analyzed thereafter in 2% agarose gel. Lanes, M_R_: RNA molecular weight marker; 1–2: IVT-mRNAs of *CARA*, naked and PTD-conjugated, respectively; 3–4: IVT-mRNAs of *CARB*, naked and PTD-conjugated, respectively.

The production of the IVT-mRNAs was conducted by in vitro transcription in parallel with poly-adenylation (Figure S2). The novel chemical reaction developed by our group was employed to covalently conjugate the selected PTD to the IVT-mRNAs (Section 2.4). As an indicator for the conjugation, a band shift assay was performed based on our previous results where a delayed transposition in gel electrophoreses of the PTD-IVT-mRNAs was recorded [42]. As shown in Figure 2, both PTD-IVT-mRNAs for *CARA* and *CARB* were characterized by a significantly retarded electrophoresis mobility compared to the respective naked IVT-mRNAs. 

### 3.2. Stability Assays of the PTD-IVT-mRNAs of CARA and CARB

**Figure 3:**
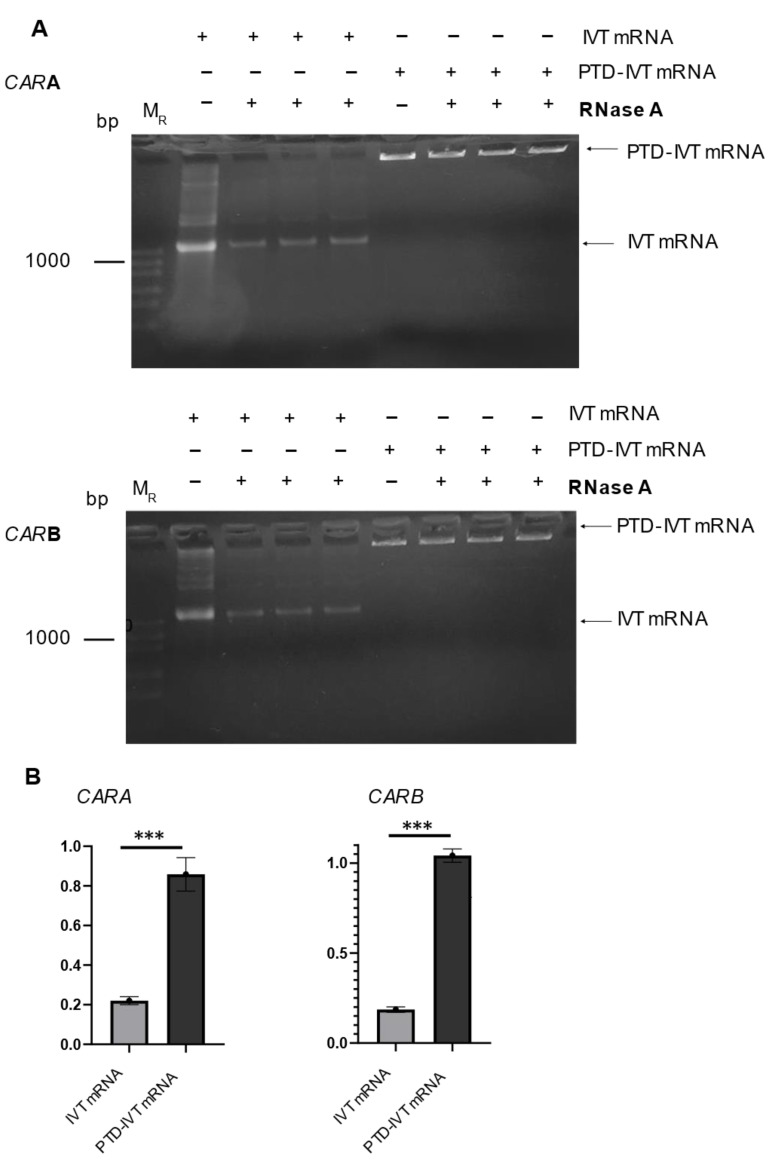
Assessment of the stability of PTD-IVT-mRNAs of *CAR* in the presence of RNase. (**A**) IVT-mRNAs as well as PTD-IVT-mRNAs were incubated in serum-free medium for 1 h at 37 °C. In a set of samples, RNase A (1 ng/mL) was also included. Next, the IVT mRNA samples were analyzed in 2% agarose gel. (**B**) The intensity of each band was quantified. The results are expressed relatively to the intensity of the bands of those IVT-mRNAs left untreated (without RNase) (set at 1.0). The same procedure was followed for PTD-IVT-mRNAs. The quantified results for both constructs (*CARA* and *CARB*) are shown. Statistically significant differences were observed between the intensity of the gel bands concerning the PTD-IVT-mRNAs vs. the intensity of gel bands from the naked IVT-mRNAs after the incubation with RNase. *p* values < 0.001 (***).

An important issue in the implementation of the IVT-mRNA technology is the stability issues in cell culture medium and/or other fluids for enough time to secure their subsequent transduction into cells and expression into the desired proteins. To examine this important concern, the PTD-IVT-mRNAs of *CARA* and *CARB* were incubated with RNase for 1 h, followed by analysis in gel electrophoresis. The same procedure was repeated for naked IVT-mRNAs (Figure 3A). Quantitative measurements of the resulting gels, concerning the construct for *CARA*, showed that only 22 ± 2% of the naked IVT-mRNA remained intact and thus detectable after the treatment with RNase (Figure 3B). On the other hand, the relevant part of the PTD-IVT-mRNA that remained intact was at 86 ± 8%. Similar results obtained for the PTD-IVT-mRNAs for *CARB* construct. The chemical conjugation of the IVT-mRNAs with the PTD significantly (<0.001) protected the IVT-mRNAs from RNase. 

### 3.3. Assessment of the Intracellular Transduction and Expression of the IVT-mRNAs of CAR into the Respective CAR Receptors, Complemented by Their Subcellular Localization in K562 Cells, Employed as a Model Cell Line

**Figure 4:**
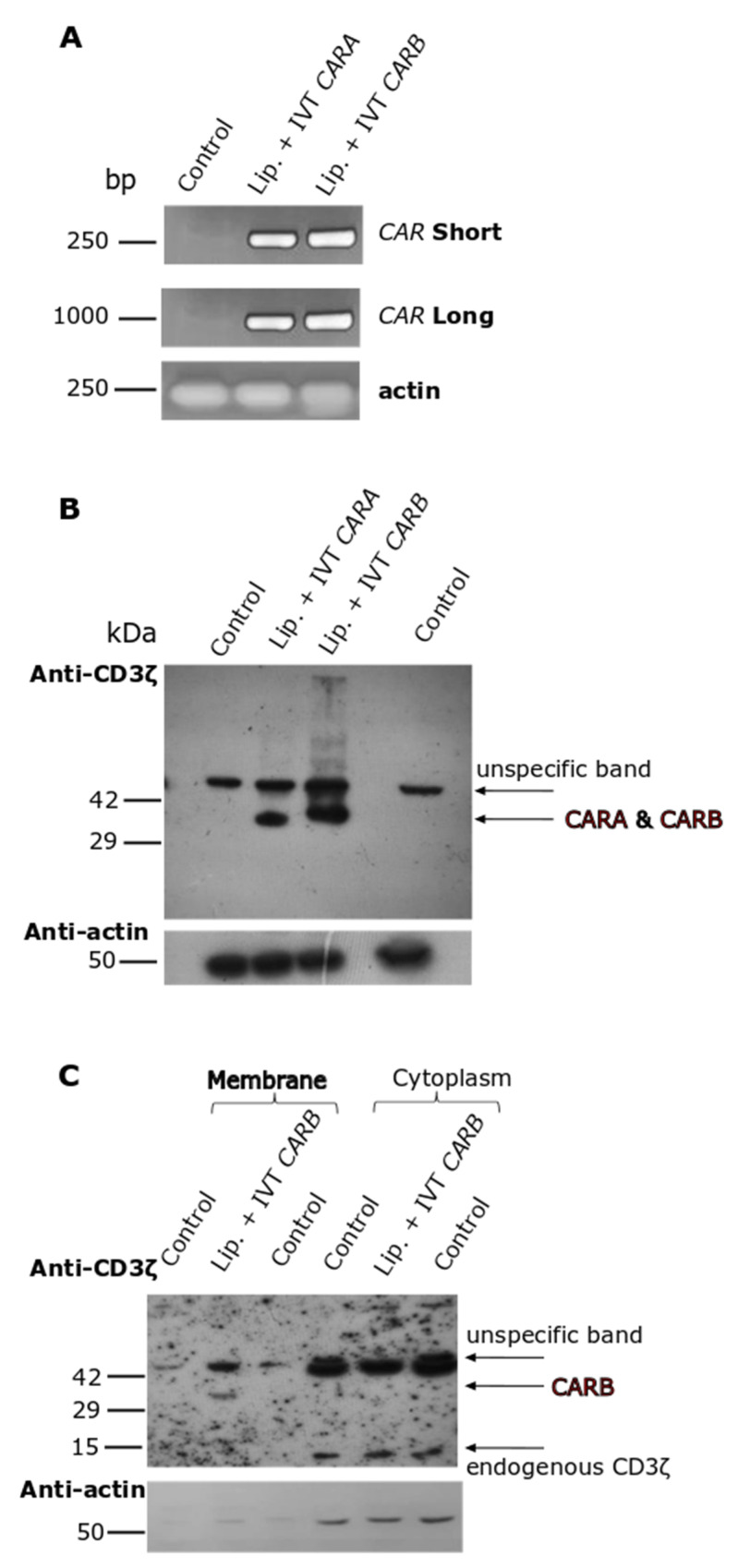
Intracellular transduction of IVT-mRNAs as well as subsequent expression and subcellular localization analysis of the corresponding CAR molecules in K562 pro-erythroid cells. (**A**) K562 cells were transfected with either IVT mRNA of *CARA* or *CARB* incubated with lipofectamine for 4 h. Thereafter, total RNA was extracted from K562 cells and RT-PCR was conducted by using two different set of primers amplifying both a part and the whole IVT-mRNA of *CAR*. Untreated K562 cells served as the negative control of transfection. *β-ACTIN* was amplified as a housekeeping gene. (**B**) K562 cells were either left untreated or transfected with IVT-mRNA of *CARA* or *CARB* incubated with lipofectamine for 24 h. Then, whole cellular extracts were prepared and analyzed by Western blotting against CD3ζ epitope. (**C**) K562 cells [transduced as described at (**B**)] were subjected to subcellular fractionation, followed again by Western blotting against CD3ζ epitope. β-actin was used as marker for equal protein loading.

In the first place, we had to explore the dynamic potential of our IVT-mRNA constructs to be expressed into the proteins and if the resulting CAR-receptors could be properly localized in the cellular membranes. Into this set of experiments, our model experimental conditions were: K562 pro-erythroid cells, an easy-to-handle cell line, transfected with the IVT-mRNAs, employing lipofectamine. PCR analysis in samples prepared by K562 cells transfected with the IVT-mRNAs of *CARA* or *CARB* in the presence of lipofectamine detected both constructs at 4 h of incubation (Figure 4A). Moreover, by using a set of primers amplifying almost the entire (96%) of the IVT-mRNA sequence, we were able to detect the full-length IVT-mRNA in our cellular samples. Then, the whole cell lysates were prepared by K562 cells transfected for 24 h to allow a sufficient time for *CAR* expression. Western blot analysis against an epitope of CD3ζ domain of *CARA* and *CARB* (Figure 1) detected both CAR-receptors at the desired length of 35 kDa (predicted molecular mass on Expasy, Swiss Bioinformatics Resource Portal) (Figure 4B). Furthermore, subcellular fractionation into membranes and cytoplasm depicted the CARB receptor to be mainly located at the membranous protein fraction (Figure 4C). Overall, our constructs were assessed by these preliminary tests for the subsequent experiments using the PTD-IVT-mRNA technology in NK-92 cells.

### 3.4. Transduction of the PTD-IVT-mRNAs of CARA and CARB in NK-92 Cells Serving as the Effector Cells

**Figure 5:**
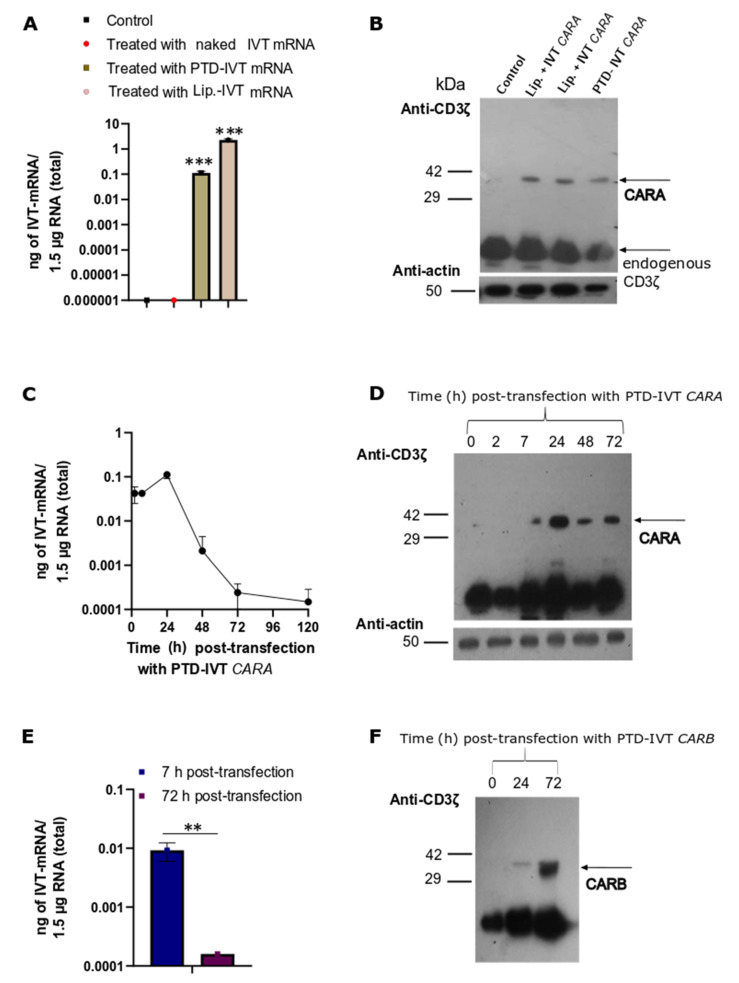
Time-dependent intracellular transduction of PTD-IVT-mRNAs of *CARA* and *CARB* and subsequent expression of *CARA* and *CARB* receptors in NK-92 cells. (**A**,**C**,**E**): (**A**) NK-92 cells were transfected with IVT-mRNA of *CARA*, either covalently conjugated to PTD or incubated with lipofectamine for 7 h. NK-92 cells treated with IVT-mRNA alone (naked) or left untreated for the same time-period (7 h) served as the negative control experiments. (**C**,**E**) NK-92 cells were transduced with PTD-IVT-mRNA of *CARA* for 2, 7, 24, 48, 72 and 96 h (**C**) or PTD-IVT-mRNA of *CARB* for 7 and 72 h (**E**). Thereafter, total RNA was extracted from cells, and cDNA was prepared, serving as the template for qPCR analysis. Based on the standard curves constructed (Figure S3), the results of qPCR analysis were expressed as ng of IVT-mRNA. (**B**,**D**,**F**): NK-92 cells were transfected (as described for the RNA extraction procedures (**A**,**C**,**E**)), and the whole cellular protein extracts were prepared at the indicated times. Then, Western blot analysis took place using an antibody against CD3ζ epitope. Statistical differences are shown pairwise (*t*-test) between the quantity of IVT-mRNA detected at NK-92 cells transduced with either PTD-IVT-mRNA for *CARA* or incubated with lipofectamine and IVT-mRNA for *CARA* and control NK-92 cells (Figure 5A), as well as the quantity of IVT-mRNA detected at 7 h and 72 h post-transduction period with PTD-IVT-mRNA of *CARB* (Figure 5E). *p* values 0.01 (**), 0.001 (***).

Our next experimental goal was to assess the efficacy of the PTD-IVT-mRNAs of *CARA* and *CARB* to be transduced and translated in the NK-92 cells. Firstly, NK-92 cells were transfected with the PTD-IVT-mRNA of *CARA* for 7 h. qPCR analysis that followed detected 0.04 ± 0.006 ng of PTD-IVT-mRNA into NK-92 cells per 1.5 μg total RNA (Figure 5A) (*p* < 0.001). Respectively, NK-92 cells transfected with naked IVT-mRNA of *CAR* thus served as our negative control experiment, showing undetectable levels. The expression analysis showed that the *CARA*-receptor was produced by the PTD-IVT-mRNA of *CARA* in the NK-92 cells at the expected molecular mass (Figure 5B). Endogenous CD3ζ was detected also at 10 kDa (NP_001365445) in both K562 and NK-92 cells (Figure 4C and Figure 5D–F); its levels were estimated irrespective of the transfection condition.

Time-course qPCR analysis in NK-92 cells transfected with the PTD-IVT-mRNA of *CARA* for different time-points (ranging from 2 to 120 h) depicted that the PTD-IVT-mRNA was accumulated early into NK-92 cells (detected at 2 h) while at 24 h reached maximum intracellular levels (at 0.10 ± 0.02 ng of PTD-IVT-mRNA) (Figure 5C). This result clearly indicated that the intracellular transport of the PTD-IVT-mRNA continued for at least 24 h. Thereafter, the intracellular levels of the PTD-IVT-mRNA started to decline almost linearly by time despite its detectable levels at 120 h post-transduction. In a parallel analysis where the protein levels of *CARA* were analyzed, the *CARA* receptor expressed with a partial delay (barely detected at 7 h) compared to the accumulation of the PTD-IVT-mRNA (Figure 5C). The protein levels of *CARA* receptor though persisted in the entire experimental timeframe (up to 72 h) (Figure 5D).

A similar conclusion was drawn for the construct of CARB-receptor, where the intracellular accumulation of the PTD-IVT-mRNA of *CARB* was decreased by >90% (*p* < 0.01) between 7 and 72 h post-transduction (Figure 5Ε). However, protein levels of CARB-receptor were highly detected at 72 h (Figure 5F). 

**Figure 6:**
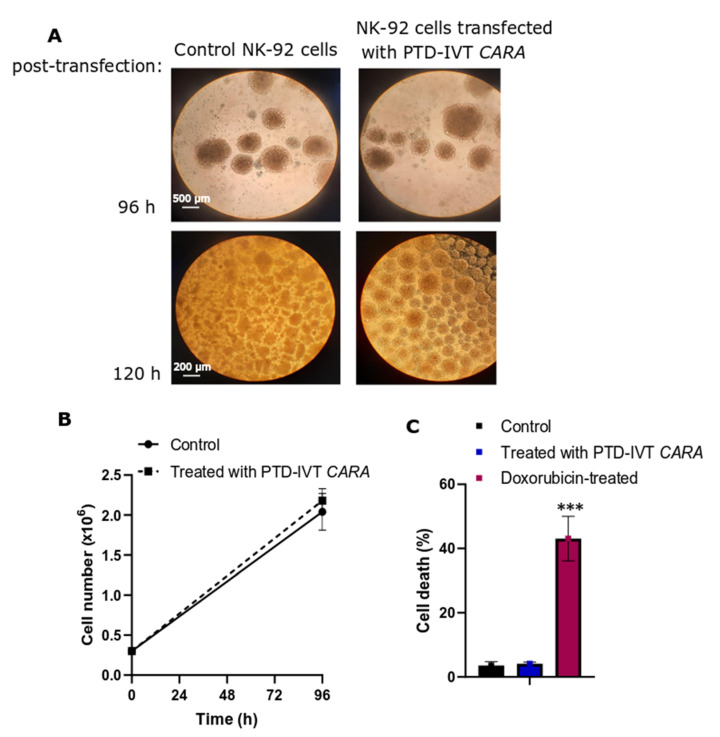
Cell morphology, growth and viability of NK-92 cells treated with the PTD-IVT-mRNA of *CARA*. (**A**) ΝΚ-92 cells were treated with PTD-IVT-mRNA of *CARA* for 96 h (magnification 4×) and 120 h (magnification 10×) and then phase-contrast photomicrographs were taken. Cells left untreated served as control experiment. (**B**,**C**) NK-92 cells were seeded at 0.3 × 10^6^ (time 0 h) in the presence and absence of PTD-IVT-mRNA of *CARA*. At 96 h, cell number was assessed by flow cytometry (**B**), while cell death was assessed by probing cells with Zombie Red as a marker of dead cells. (**C**) NK-92 cells treated with doxorubicin (2 μM) for 72 h were used as a positive sample for cell death. ANOVA test: *p* = 0.000031 (significant); pairwise *t*-test: control-treated with PTD-IVT-mRNA of *CARA*, *p* = 0.51 (not significant); pairwise *t*-test: control-doxorubicin-treated, *p* = 0.00064. *p* values 0.001 (***).

Before we proceed to the co-incubation assays with the ErbB+ cancer cells, we examined if the transfection with PTD-IVT-mRNAs of *CARA* and *CARB* has negative impact on cell growth and viability of NK-92 cells. Incubation with PTD-IVT-mRNA of *CARA* depicted no morphological alterations as assessed by optical microcopy (Figure 6A); cellular ‘’batches’’ formed by NK-92 cells at their regular growth can be observed in Figure 6A, in both NK-92 cells left untreated and those transfected with PTD-IVT-mRNA of *CARA* for up to 120 h. According to our results, the transfection of NK-92 cells with PTD-IVT-mRNA did not result in impaired cell growth (Figure 6B) or cell damage (Figure 6C), permitting us to carry on with the co-incubation experiments.

### 3.5. Cytotoxic Potential of the PTD-IVT-mRNA-Engineered CAR-NK-92 Cells on Oral Squamous HSC-3 Cells

NK-92 cells recognize the CD52 and CD102 markers of major histocompatibility complex on the surface of K562 cells [58] leading to their granule-mediated apoptosis [59]. A CAR-receptor-independent co-incubation cytotoxicity assay was set to assess the cytotoxic potential of NK-92 cells against the K562 cells. NK-92 cells (untransduced) were co-incubated with K562 cells (E:T ratio at 10:1) for 6 h. Cell death was assessed at 35 ± 2% at the co-culture of E:T cells, compared to NK-92 cells or/and K562 cells cultured separately (cell death at less than 3%) (Figure S4).To further enhance the cytotoxic potential of NK-92 cells, the chemical compound 2,3-butanediol was added at NK-92 cell cultures; 2,3-butanediol induces the perforin expression of NK-92 cells, and by doing so, their cytotoxic activity [56]. Moreover, no negative impact on cell growth of NK-92 cells was recorded in NK-92 cell cultures-treated with 2,3-butanediol, (Figure S5), both in the presence and absence of the PTD-IVT-mRNAs. By this time, 2,3-butanediol was regularly added at NK-92 cell cultures during the transduction period with the PTD-IVT-mRNA, and then proceeded with the co-incubation assays with the ErbB+ targeted cancer cell lines (see Section 2.9).

**Figure 7:**
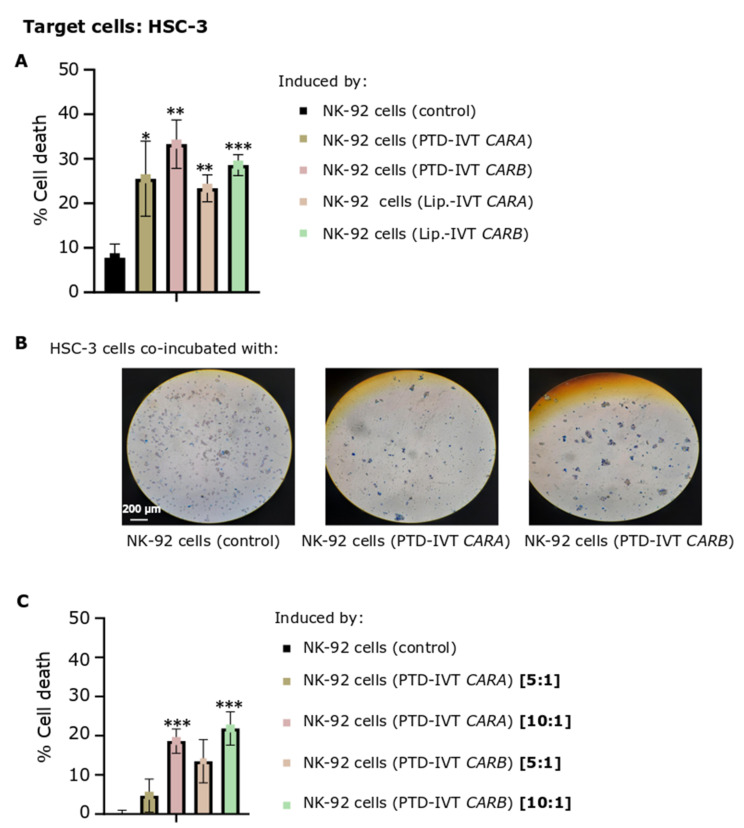
Cytotoxic potential of PTD-IVT-mRNA-engineered CAR-NK-92 cells either expressing *CARA* or CARB receptor on HSC-3 oral squamous cells. (**A**) Percentage of cell death of HSC-3 cells (T) induced by the co-incubation with NK-92 cells engineered with either the PTD-IVT-mRNAs or Lipοfectamine (Lip.)-IVT-mRNAs of *CARA* and *CARB*(E), as well as percentage of cell death of HSC-3 cells induced by control (untransduced) NK-92 cells. NK-92 cells were transfected with either PTD-IVT-mRNA and then co-incubated with HSC-3 cells, as described in Section 2.9, at a ratio of 10:1 (E:T). (**B**) Representative optical microscopy images of trypan-blue-stained, co-incubated cellular populations, as depicted in each image (magnification ×10). (**C**) Percentage of cell death of HSC-3 cells induced by the co-incubation with PTD-IVT-mRNAs of *CARA* and CARB-engineered NK-92 cells, mixed at two different ratios of E:T cells, either 5:1 or 10:1. Statistical differences are shown between cells co-incubated with engineered NK-92 cells compared to cell death induced by control NK-92 cells, as shown in its graph. *p* values < 0.05 (*), 0.01 (**), 0.001 (***). ANOVA test: *p* = 0.001 (Figure 7A), *p* = 0.003 (Figure 7C).

The efficacy of CAR-dependent NK-92 cell-induced cytotoxicity was next assessed on HSC-3 OSCC cells expressing the ErbB receptors at quite high levels [60]. ΝΚ-92 cells were transduced with the PTD-IVT-mRNAs of *CARA* or *CARB* for 36 h, a time-period of transduction selected based on the time-course accumulation of CAR-receptors post-transduction (Figure 5D). NK-92 cells engineered with the PTD-IVT-mRNAs were then co-incubated with HSC-3 cells at E:T ratios of 5:1 to 10:1. As shown at Figure 7A, the proportion of dead HSC-3 cells induced by the co-incubation with NK-92 cells transduced with the PTD-IVT-mRNA of *CARA* and *CARB* was estimated at 25% ± 8 (*p* < 0.05) and 33% ± 5 (*p* < 0.01), respectively, at the ratio of E:T at 10:1. Comparatively, cell death of HSC-3 cells co-incubated with untransduced NK-92 cells was recorded at 8% ± 3. HSC-3 cultures co-incubated with NK-92 cells transduced in the presence of lipofectamine with the respective IVT-mRNAs were characterized by a similar proportion of dead cells (Figure 7A) as in the case of the PTD-mediated transduction of NK-92 cells. Indicative optical microscopy images of trypan blue-stained HSC-3 cells on the different co-incubation treatments are shown in Figure 7B. Moreover, the CAR-NK-92 cell-induced cytotoxicity was dose-dependent in the ratios of E:T ranging between 5:1 to 10:1 (Figure 7C). The percentages of cell death of HSC-3 cells cultured separately as control-cultures are illustrated in Figures S6 and S7. Moreover, ΝΚ-92 cells incubated in separate cultures in each experimental state (untransduced, transduced with PTD-IVT-mRNA of *CAR* and/or transduced with IVT-mRNA of *CAR* in presence of lipofectamine) were assessed for cell death proportion irrespective of the co-incubation with HSC-3 cells (also shown in Figures S6 and S7). 

**Figure 8:**
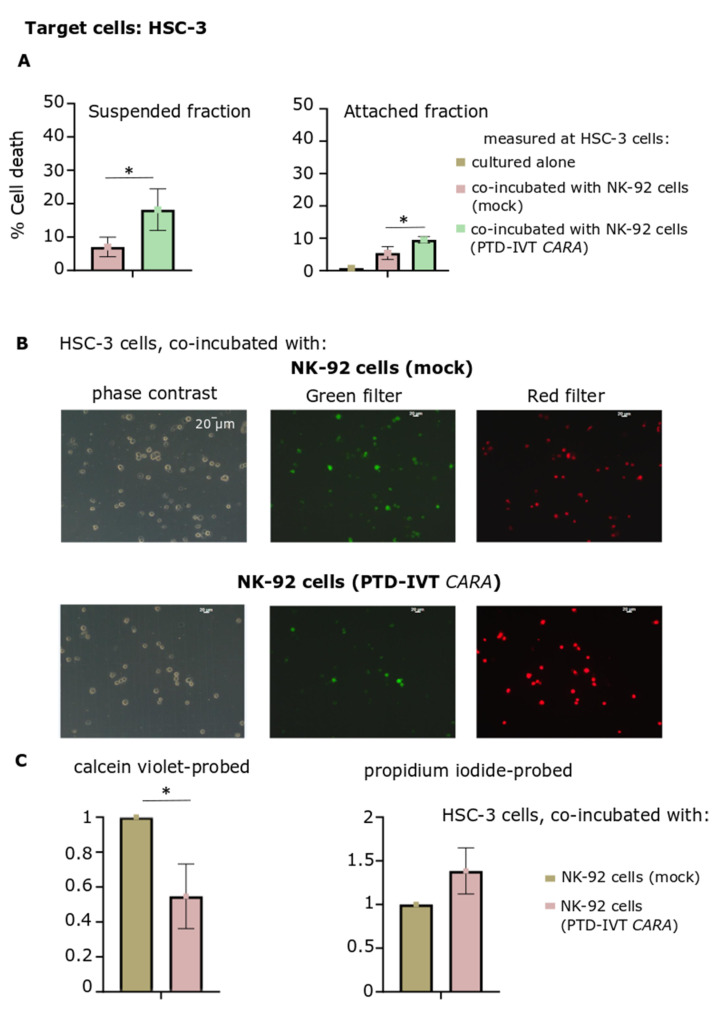
Assessment of cytotoxicity of NK-92 cells engineered with PTD-IVT-mRNA of *CARA* on HSC-3 cells by cell death measurement on suspended and attached fractions of the co-cultures as well as by fluorescent microscopy. (**A**) Cell death percentage at suspended and attached fractions in the co-incubation cultures of HSC-3 cells with NK-92 cells, either transduced with the PTD-IVT-mRNA of *CARA* or control ones. NK-92 cells were either remained untreated or transduced with the PTD-IVT-mRNA of *CARA* and co-incubated with HSC-3 cells (ratio 10:1, E:T), as described in Section 2.9. Afterwards, for each co-incubation treatment, cells remaining in culture medium in suspended form were removed in a separate tube after soft handling, yielding the suspended fraction. Cells remained attached were trypsinized, being the attached fraction of each design. Each fraction of cells was assessed for cell death by trypan blue exclusion assay, separately. The cell death percentages measured are presented, along with those of HSC-3 cells, cultured separately. (**B**) After the co-incubation period of E:T cells (10:1), cells (both suspended and attached) of each experimental design were collected in the same tube and proceeded for probing with both calcein violet and propidium iodide (Section 2.10). Representative images in the phase contrast, green and red filters are presented. (**C**) The quantified results of (**B**) are shown. The intensity was normalized relative to the images acquired by HSC-3 cells co-incubated with NK-92 cells (control) (set at 1). The same procedure was repeated for both calcein violet and propidium iodide fluorescent microscopy images. Statistical significance is shown between HSC-3 cells co-incubated with NK-92 cells transfected with PTD-IVT-mRNA of *CARA* and those HSC-3 cells co-incubated with NK-92 cells left untreated [*p* value < 0.05 (*)].

Subsequently, an alternative approach was followed, where cell death of HSC-3 cells induced by the engineered NK-92 cells was assessed in two separate fractions after the co-incubation period: in cells remaining attached in culture and those cells being suspended in culture medium (Figure 8A). PTD-IVT-mRNA of *CARA* was indicatively selected to be used for these transfection experiments with NK-92 cells. Both cellular fractions (attached and suspended ones) were characterized by higher proportions of dead HSC-3 cells (*p* < 0.05) provoked by CAR-engineered NK-92 cells compared to the untransduced NK-92 cells.

Finally, cultures of HSC-3 cells co-incubated with NK-92 cells either transduced with PTD-IVT-mRNA of *CARA* or untreated ones were assessed for cell viability and death by fluorescent microscopy. Cells were probed with both calcein violet and propidium iodide, as indicators of viable and dead cells, respectively (Figure 8B). Quantitation of the fluorescent microscopy images depicted reduction of viable cells by 50% (at 0.5 ± 0.2, *p* < 0.05) at HSC-3 cells co-incubated with PTD-IVT-mRNA-engineered CAR-NK-92 cells. At the same time, propidium iodide-stained cells increased by 38% (Figure 8C). To sum up, the PTD-IVT-mRNA conjugates were properly transduced into NK-92 cells, translating into the corresponding CAR receptors and leading to functional effector cells in the co-incubation assays with ErbB+ HSC-3 cells.

### 3.6. MCF-7 Breast Metastatic Adenocarcinoma Cells Expressed Significantly Lower ErbB2 Receptors but Were Targeted and Killed by the PTD-IVT-mRNA-Engineered CAR-NK-92 Cells to almost the Same Levels with HSC-3 OSCC Cells

**Figure 9:**
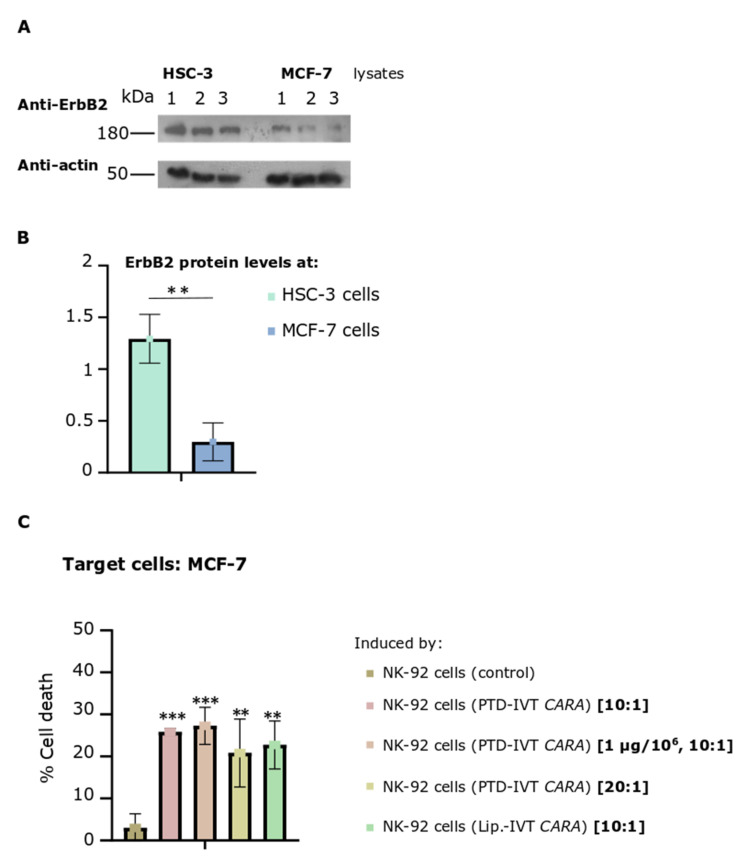
NK-92 cells (transfected via PTD-IVT-mRNA of *CARA* to express *CARA* receptor)-induced cytotoxicity on MCF-7 breast adenocarcinoma cells. (**A**,**B**) ErbB2 protein levels of HSC-3 and MCF-7 cells. (**A**) HSC-3 or MCF-7 cells were seeded at 5 × 10^4^ cells/cm^2^ and left to grow for 72 h, when extraction of proteins took place. The protein lysates were analyzed by Western blotting for ErbB2. β-actin was used as a protein loading control. Three different biological replicates are shown for each cell line. (**B**) The intensity of the ErbB2 proteins bands of each sample was quantified and then normalized to β-actin. Statistical significance of the expression of ErbB2 receptor between the two cell lines is shown (*p* < 0.01). (**C**) MCF-7 cells were co-incubated with NK-92 cells, previously transfected with PTD-IVT-mRNA of *CARA* in the default transfection conditions (0.5 μg/10^6^ NK-92 cells) or at 1 μg/10^6^ NK-92 cells. Two different ratios of E:T cells (10:1 to 20:1) were tested thereafter. The transfection and co-incubation periods as well as the procedure to assess cell death are described in Section 2.9. MCF-7 cells co-incubated with NK-92 cells (control) or transfected with lipofectamine-IVT mRNA were also included. The raw results of the entire experimental treatment are presented in Figure S8. Statistical significance is shown between MCF cells co-incubated with NK-92 (control) and between those MCF-7 cells co-incubated with those transduced via the PTD-IVT-mRNA *CARA*-NK-92 cells (*p* values < 0.01 (**), 0.001 (***)). ANOVA test: *p* = 0.001.

In the last part, our study was extended by assessing the cytotoxic efficacy of the PTD-IVT-mRNA-engineered NK-92 cells expressing the *CARA* receptor to kill the MCF-7 breast adenocarcinoma cells. The expression levels of the ErbB2-receptor in MCF-7 cells were assessed by Western blot analysis complemented by quantitative measurements (Figure 9A,B). MCF-7 cells were found to express the ErbB2 receptor at around 23% (*p* < 0.01) compared to its protein levels at HSC-3 cells (Figure 9B).

The co-incubation of MCF-7 cells with PTD-IVT-mRNA-engineered *CARA*-NK-92 cells resulted in 26% ± 3 cell death compared to 3% ± 3 (*p* < 0.001) of control NK-92 cells (Figure 9C). Having already this promising result, we sought to raise the percentage of CAR-NK-92-cell-mediated cell death of MCF-7 cells by either increasing the ratio of E:T from 10:1 to 20:1, or while the ratio of E:T remained stable at 10:1, NK-92 cells were transfected with 1 μg instead of 0.5 μg of PTD-IVT-mRNA of *CARA*. NK-92-mediated cytotoxicity was recorded at 27 ± 4 and 21 ± 8 (*p* < 0.01) in these differentiated conditions concerning the E:T ratio and transduction parameters, respectively.

## 4. Discussion

CAR immunotherapy yields spectacular therapeutic results in treating leukemias, such as ALL, since the first patients treated with this innovative therapy are disease-free several years later [61]. The main approach involves using ex vivo genetically modified by viral vectors, T cells from patient’s blood. Viral transfection is the main way to transduce the *CAR* construct [24]. 

However, the application of CAR immunotherapy for the treatment of solid tumors is still being developed. Several problems, such as the CAR T cell exhaustion in the tumor microenvironment and the difficulty in selecting the proper TAAs [62] for targeting the malignant cells, are significant drawbacks. Moreover, the low yield isolation of autologous T cells from cancer-suffering patients are a major obstacle; to this end, other sources of immune cells have been proposed to ideally construct universal CAR therapies. 

Currently, numerous CAR immunotherapy programs are under development by approximately 140 pharmaceutical/biotech companies. The cost of such therapies is extremely high since one single infusion costs USD 475,000 [63]. In addition, two ongoing clinical studies in patients with advanced stage breast cancer employ CAR T cells targeting ErbB2 (Human Epidermal growth factor Receptor 2, HER2) and mesothelin [64]. However, in some participants, toxicity and subsequent CRS leading to fatal organ failure have been recorded. In head and neck cancer, clinical trials employing CAR T cells with T1E scFv (PanErbB) applied locally in cancer lesions are ongoing [65].

In the present work, a novel approach of CAR immunotherapy is being investigated, where the CDS of *CAR* is transduced into NK-92 cells in the form of an IVT-mRNA[40] through an innovative delivery method [42] designed by our group. This method employed the covalent linkage of the selected PTD to the IVT-mRNA of *CAR* for transduction into mammalian cells towards the expression of the desired protein and gaining of the relevant function. NK-92 cells are a homogenous cell line of effective immune cells that overcome the graft-host reactions. Designed CARs recognize the ErbB-receptors, which are highly expressed TAAs in solid tumors. 

The sequence of T1E scFv was used to construct two CAR molecules containing CD28 or 4-1BB as the co-stimulatory domains. Thereafter, IVT-mRNAs with appropriate 5′- and 3′-UTRs, translation initiation sequences and poly-A tails were then produced. The IVT-mRNAs were covalently conjugated to the selected PTD via our patented chemical reaction [42]. Prior to use, stability assays of the PTD-IVT-mRNAs as well as preliminary transduction experiments in K562 cells were performed. Both PTD-IVT-mRNAs were rapidly transduced and expressed in corresponding CARA and CARB receptors into NK-92 cells, being the effector cells. PTD-IVT-mRNA-engineered CAR-NK-92 cells were grown robustly vs. control NK-92 cells. In co-incubation experiments, two different ErbB+ cancer cells were recruited, the HSC-3 and the MCF-7 cells, derived from solid malignant tumors. The CAR-NK-92-cell-mediated cytotoxicity was assessed in attached and detached cells. In addition, fluorescent microscopy was performed by using indicative probes of cell viability and death. The ratios of Effector (E):Target (T) cells were between 5:1 and 20:1. 

PTDs have been successfully used over the years for protein replacement therapy (PRT) [43,44,46]. Mechanistically, PTDs transduce cellular membranes via multiple mechanisms, such as endocytosis, direct penetration and others, to facilitate the internalizations of variant cargoes that carry on inside the cells [66,67,68,69]. In our delivery system, clathrin-mediated endocytosis was found to take place since its specified inhibitor, chlorpromazine, almost entirely blocked the intracellular accumulation of the studied PTD-IVT-mRNA [42].

Conformational alterations in the PTD-IVT-mRNAs, assessed by NMR analysis [42], possibly contribute to their higher protection from the RNase action compared to the corresponding naked IVT-mRNAs (Figure 3). The PTD-IVT-mRNAs were transduced into both cell lines employed, K562 (Figure 4A,B) and NK-92 (Figure 5A,C,E) cells. Based on our results, we can confirm that transduced PTD-IVT-mRNAs remained relatively stable in culture for at least 24 h considering that the intracellular accumulation increased between 2 and 24 h post-transduction (Figure 5C). Thereafter, between 24 and 120 h, a linear decline in intracellular levels of PTD-IVT-mRNAs was observed, which could be explained by the enzymatic degradation of PTD-IVT-mRNAs and/or dilution due to cell division. The kinetics of the intracellular accumulation of PTD-IVT-mRNAs is consistent with previous studies employing IVT-mRNAs and alternative transduction systems [70]. 

Comparatively to the intracellular accumulation of the PTD-IVT-mRNAs, the protein expression of the corresponding CAR receptors persisted up to 72 h post-transduction (Figure 5D,F), a consistent finding with our previous study [42]. Many factors may contribute to the steady-state levels of a protein produced by a transduced IVT-mRNA, such as the translational efficiency of the IVT-mRNAs, the stability of ribonucleoprotein complexes formed as well the half-life of the protein itself [71]. In our previous study, mitochondrial Sco2 protein produced by the PTD-IVT-mRNA of *SCO2* was found to be functional, as assessed via a histochemical staining of the cytochrome c oxidase (COX) activity in primary fibroblasts derived from an *SCO2/COX*-deficient patient [42]. Both CAR molecules were able to bind to ErbB receptors on the surface of target cells, as assessed by the CAR-NK-92-mediated cytotoxicity in both HSC-3 cells (Figure 7 and Figure 8) and MCF-7 cells (Figure 9). NK-92 cells, being activated by the co-stimulatory intracellular sequences, lead to the secretion of death molecules (perforins, granzymes), as well activation of death receptor signaling via Fas/Fas-ligand (Fas-L) or TNF/TNF-R, in the lytic synapses with their target T cells [72].

The ErbB2 levels in oral cancer have been proposed as a molecular marker for disease prognosis [73]. In clinical therapeutics, Trastuzumab, a humanized monoclonal antibody targeting cells overexpressed ErbB2 (HER2), is administered mainly in HER2-positive breast cancer, offering survival advantage in these patients [74]. Moreover, ErbB1 (epidermal growth factor receptor, EGFR) is frequently over-expressed in OSCC, and as so, monoclonal antibodies (as cetuximab) in combination with small molecular inhibitors are already used in therapy [75]. However, the ErbB network of receptors is more complicated than this, and it is nowadays believed that the ErbB protein network plays a fundamental role in divergent solid tumor progression [76,77,78]. In our experimental procedure, the PTD-IVT-mRNAs-engineered CAR-NK-92 cells induced comparable cytotoxicity on both ErbB+ cancer cell lines tested (Figure 7 and Figure 9B).

MCF-7 (malignant breast cell carcinoma) cells expressed relatively low levels of the ErbB2 receptor compared to levels expressed in HSC-3 cells (Figure 9A). It is worth mentioning that the T1E scFv is activated by different dimers of ErbB receptors, such as the ErbB1 homodimers and the ErbB2/3 heterodimer [50]. As such, multifunctional targeting of the ErbB network of receptors is achieved by employing the T1E scFV sequence in CAR-NK-92 cells [50]. 

In the present study, two constructs of second generation CAR-receptors were tested, differing in the presence of the co-stimulatory domain that was either the CD28 (*CARA*) or the 4-1BB (*CARB*). Both constructs displayed comparable rates of cytotoxicity while there was a tendency for higher cytotoxicity levels induced by CARB-NK-92 cells (Figure 7A,B). According to Kawalekar et al., 4-1BB offers greater proliferation and persistence than central memory cells do over CD28 CARΤ-cells [5,18]. In the same study, CAR T cells bearing CD28 were found to use aerobic glycolysis, while CAR T cells having the 4-1BB co-stimulatory domain made use of oxidative breakdown of fatty acids for energy production. The study of the different co-stimulatory sequences on the bioenergetics of the CAR-NK-92 cells is under investigation by our research group. 

CAR-mediated targeting of cancer cells leading to their apoptotic death is a highly desirable therapeutic approach in cancer. Future studies should investigate the efficacy of the PTD-IVT-mRNA-engineered CAR-NK-92 cells in preclinical models [79]. Colony forming assays will give further merit to our work evaluating the clonogenicity of cancer cells remnant from CAR-NK-92-cell-mediated cytotoxicity [80]. 

In conclusion, this study contributes to the field of CAR immunotherapy by applying our innovative PTD-IVT-mRNA delivery platform in the transduction of a highly promiscuous, multifunctional IVT-mRNA of *CAR* into NK-92 cells as a homogenous off-the shelf immune cell population.

## Data Availability

All the data associated with the study are presented in the main article and/or in the Supplementary Materials.

## Supplementary Materials

The following supporting information can be downloaded at: https://www.mdpi.com/article/10.3390/biomedicines10112885/s1, Figure S1. Assessment of cloning of *CARA* and *CARB* inserts into pGEM vector via proper, sense orientation for in vitro transcription; Figure S2. Electrophoresis mobility pattern of *CARA* and *CARB* IVT-mRNAs; Figure S3. Standard Curves of PTD-IVT-mRNAs for *CARA* and *CARB;* Figure S4. Assessment of NK-92-cell-induced cytotoxicity on K562 cells; Figure S5. Cell growth of NK-92 cells treated with 2,3-butanediol in the presence and absence of PTD-IVT-mRNA of *CARA*; Figure S6. Cell death percentage of the different experimental treatments of HSC-3 and/or NK-92 cells (engineered or not) cultured separately or co-incubated simultaneously; Figure S7. Cell death (raw and calculated results), presented in Figure 7C; Figure S8. The raw results of the NK-92-induced cytotoxicity on MCF-7 cells, as presented in Figure 9C.

## References

[1] Dunn G.P., Old L.J., Schreiber R.D. (2004). The three Es of cancer immunoediting. Annu. Rev. Immunol..

[2] Gross G., Waks T., Eshhar Z. (1989). Expression of immunoglobulin-T-cell receptor chimeric molecules as functional receptors with antibody-type specificity. Proc. Natl. Acad. Sci. USA.

[3] Miliotou A.N., Papadopoulou L.C. (2018). CAR T-cell Therapy: A New Era in Cancer Immunotherapy. Curr. Pharm. Biotechnol..

[4] Singh N., Frey N.V., Grupp S.A., Maude S.L. (2016). CAR T Cell Therapy in Acute Lymphoblastic Leukemia and Potential for Chronic Lymphocytic Leukemia. Curr. Treat. Options Oncol..

[5] Kawalekar O.U., O’Connor R.S., Fraietta J.A., Guo L., McGettigan S.E., Posey A.D., Patel P.R., Guedan S., Scholler J., Keith B. (2016). Distinct Signaling of Coreceptors Regulates Specific Metabolism Pathways and Impacts Memory Development in CAR T Cells. Immunity.

[6] Xu X., Huang S., Xiao X., Sun Q., Liang X., Chen S., Zhao Z., Huo Z., Tu S., Li Y. (2020). Challenges and Clinical Strategies of CAR T-Cell Therapy for Acute Lymphoblastic Leukemia: Overview and Developments. Front. Immunol..

[7] Maakaron J.E., Hu M., El Jurdi N. (2022). Chimeric antigen receptor T cell therapy for cancer: Clinical applications and practical considerations. BMJ.

[8] Cao B., Liu M., Wang L., Liang B., Feng Y., Chen X., Shi Y., Zhang J., Ye X., Tian Y. (2020). Use of chimeric antigen receptor NK-92 cells to target mesothelin in ovarian cancer. Biochem. Biophys. Res. Commun..

[9] Munisvaradass R., Kumar S., Govindasamy C., Alnumair K.S., Mok P.L. (2017). Human CD3+ T-Cells with the Anti-ERBB2 Chimeric Antigen Receptor Exhibit Efficient Targeting and Induce Apoptosis in ERBB2 Overexpressing Breast Cancer Cells. Int. J. Mol. Sci..

[10] Zhong X.-S., Matsushita M., Plotkin J., Riviere I., Sadelain M. (2010). Chimeric antigen receptors combining 4-1BB and CD28 signaling domains augment PI3kinase/AKT/Bcl-XL activation and CD8+ T cell-mediated tumor eradication. Mol. Ther..

[11] Thaci B., Brown C.E., Binello E., Werbaneth K., Sampath P., Sengupta S. (2014). Significance of interleukin-13 receptor alpha 2-targeted glioblastoma therapy. Neuro Oncol..

[12] Li J., Li W., Huang K., Zhang Y., Kupfer G., Zhao Q. (2018). Chimeric antigen receptor T cell (CAR-T) immunotherapy for solid tumors: Lessons learned and strategies for moving forward. J. Hematol. Oncol..

[13] Jung M., Yang Y., McCloskey J.E., Zaman M., Vedvyas Y., Zhang X., Stefanova D., Gray K.D., Min I.M., Zarnegar R. (2020). Chimeric Antigen Receptor T Cell Therapy Targeting ICAM-1 in Gastric Cancer. Mol. Ther. Oncolytics..

[14] Wang Y., Chen M., Wu Z., Tong C., Dai H., Guo Y., Liu Y., Huang J., Lv H., Luo C. (2018). CD133-directed CAR T cells for advanced metastasis malignancies: A phase I trial. OncoImmunology.

[15] Liu M., Wang X., Li W., Yu X., Flores-Villanueva P., Xu-Monette Z.Y., Li L., Zhang M., Young K.H., Ma X. (2020). Targeting PD-L1 in non-small cell lung cancer using CAR T cells. Oncogenesis.

[16] Batra S.A., Rathi P., Guo L., Courtney A.N., Fleurence J., Balzeau J., Shaik R.S., Nguyen T.P., Wu M.-F., Bulsara S. (2020). Glypican-3–Specific CAR T Cells Coexpressing IL15 and IL21 Have Superior Expansion and Antitumor Activity against Hepatocellular Carcinoma. Cancer Immunol. Res..

[17] Marofi F., Motavalli R., Safonov V.A., Thangavelu L., Yumashev A.V., Alexander M., Shomali N., Chartrand M.S., Pathak Y., Jarahian M. (2021). CAR T cells in solid tumors: Challenges and opportunities. Stem Cell Res. Ther..

[18] Gumber D., Wang L.D. (2022). Improving CAR-T immunotherapy: Overcoming the challenges of T cell exhaustion. eBioMedicine.

[19] Hoskin D.W., Mader J.S., Furlong S.J., Conrad D.M., Blay J. (2008). Inhibition of T cell and natural killer cell function by adenosine and its contribution to immune evasion by tumor cells (Review). Int. J. Oncol..

[20] Liu Y., Fang Y., Chen X., Wang Z., Liang X., Zhang T., Liu M., Zhou N., Lv J., Tang K. (2020). Gasdermin E-mediated target cell pyroptosis by CAR T cells triggers cytokine release syndrome. Sci. Immunol..

[21] Rice J., Nagle S., Randall J., Hinson H.E. (2019). Chimeric Antigen Receptor T Cell-Related Neurotoxicity: Mechanisms, Clinical Presentation, and Approach to Treatment. Curr. Treat Options Neurol..

[22] Ruella M., Kenderian S.S. (2017). Next-Generation Chimeric Antigen Receptor T-Cell Therapy: Going off the Shelf. BioDrugs.

[23] Neelapu S.S., Tummala S., Kebriaei P., Wierda W., Gutierrez C., Locke F.L., Komanduri K.V., Lin Y., Jain N., Daver N. (2018). Chimeric antigen receptor T-cell therapy—Assessment and management of toxicities. Nat. Rev. Clin. Oncol..

[24] Michels A., Ho N., Buchholz C.J. (2022). Precision medicine: In vivo CAR therapy as a showcase for receptor-targeted vector platforms. Mol. Ther..

[25] Sun Z., Li R., Shen Y., Tan S., Ding N., Xu R., Wang X., Wei J., Liu B., Meng F. (2022). In situ antigen modification-based target-redirected universal chimeric antigen receptor T (TRUE CAR-T) cell therapy in solid tumors. J. Hematol. Oncol..

[26] Biederstädt A., Rezvani K. (2021). Engineering the next generation of CAR-NK immunotherapies. Int. J. Hematol..

[27] Klingemann H., Boissel L., Toneguzzo F. (2016). Natural Killer Cells for Immunotherapy—Advantages of the NK-92 Cell Line over Blood NK Cells. Front. Immunol..

[28] Zhang C., Oberoi P., Oelsner S., Waldmann A., Lindner A., Tonn T., Wels W.S. (2017). Chimeric Antigen Receptor-Engineered NK-92 Cells: An Off-the-Shelf Cellular Therapeutic for Targeted Elimination of Cancer Cells and Induction of Protective Antitumor Immunity. Front. Immunol..

[29] Khawar M.B., Sun H. (2021). CAR-NK Cells: From Natural Basis to Design for Kill. Front. Immunol..

[30] Sabbah M., Jondreville L., Lacan C., Norol F., Vieillard V., Roos-Weil D., Nguyen S. (2022). CAR-NK Cells: A Chimeric Hope or a Promising Therapy?. Cancers.

[31] Suerth J.D., Schambach A., Baum C. (2012). Genetic modification of lymphocytes by retrovirus-based vectors. Curr. Opin. Immunol..

[32] Miliotou A.N., Papadopoulou L.C. (2020). In Vitro-Transcribed(IVT)-mRNA CAR Therapy Development. Methods Mol. Biol..

[33] Sergeeva O.V., Koteliansky V.E., Zatsepin T.S. (2016). mRNA-Based Therapeutics—Advances and Perspectives. Biochemistry.

[34] Grudzien-Nogalska E., Stepinski J., Jemielity J., Zuberek J., Stolarski R., Rhoads R.E., Darzynkiewicz E. (2007). Synthesis of Anti-Reverse Cap Analogs (ARCAs) and their Applications in mRNA Translation and Stability. Methods Enzymol..

[35] Andries O., Mc Cafferty S., De Smedt S.C., Weiss R., Sanders N.N., Kitada T. (2015). N1-methylpseudouridine-incorporated mRNA outperforms pseudouridine-incorporated mRNA by providing enhanced protein expression and reduced immunogenicity in mammalian cell lines and mice. J. Control. Release.

[36] Kariko K., Buckstein M., Ni H., Weissman D. (2005). Suppression of RNA recognition by Toll-like receptors: The impact of nucleoside modification and the evolutionary origin of RNA. Immunity.

[37] Ross J., Sullivan T.D. (1985). Half-lives of beta and gamma globin messenger RNAs and of protein synthetic capacity in cultured human reticulocytes. Blood.

[38] Broderick K.E., Humeau L.M. (2015). Electroporation-enhanced delivery of nucleic acid vaccines. Expert Rev. Vaccines.

[39] Okur N.U., Siafaka P.I., Gokce E.H. (2021). Challenges in Oral Drug Delivery and Applications of Lipid Nanoparticles as Potent Oral Drug Carriers for Managing Cardiovascular Risk Factors. Curr. Pharm. Biotechnol..

[40] Beck J.D., Reidenbach D., Salomon N., Sahin U., Türeci Ö., Vormehr M., Kranz L.M. (2021). mRNA therapeutics in cancer immunotherapy. Mol. Cancer.

[41] Brasseur R., Divita G. (2010). Happy birthday cell penetrating peptides: Already 20 years. Biochim. Biophys. Acta.

[42] Miliotou A.N., Pappas I.S., Spyroulias G., Vlachaki E., Tsiftsoglou A.S., Vizirianakis I.S., Papadopoulou L.C. (2021). Development of a novel PTD-mediated IVT-mRNA delivery platform for potential protein replacement therapy of metabolic/genetic disorders. Mol. Ther. Nucleic Acids.

[43] Miliotou A.N., Papagiannopoulou D., Vlachaki E., Samiotaki M., Laspa D., Theodoridou S., Tsiftsoglou A.S. (2021). PTD-mediated delivery of alpha-globin chain into Kappa-562 erythroleukemia cells and alpha-thalassemic (HBH) patients’ RBCs ex vivo in the frame of Protein Replacement Therapy. J. Biol. Res..

[44] Kaiafas G.C., Papagiannopoulou D., Miliotou A.N., Tsingotjidou A.S., Chalkidou P.C., Tsika A.C., Spyroulias G.A., Tsiftsoglou A.S., Papadopoulou L.C. (2020). In vivo biodistribution study of TAT-L-Sco2 fusion protein, developed as protein therapeutic for mitochondrial disorders attributed to SCO2 mutations. Mol. Genet Metab Rep..

[45] Papadopoulou L.C., Ingendoh-Tsakmakidis A., Mpoutoureli C.N., Tzikalou L.D., Spyridou E.D., Gavriilidis G.I., Kaiafas G.C., Ntaska A.T., Vlachaki E., Panayotou G. (2018). Production and Transduction of a Human Recombinant beta-Globin Chain into Proerythroid K-562 Cells To Replace Missing Endogenous beta-Globin. Mol. Pharm..

[46] Foltopoulou P.F., Tsiftsoglou A.S., Bonovolias I.D., Ingendoh A.T., Papadopoulou L.C. (2010). Intracellular delivery of full length recombinant human mitochondrial L-Sco2 protein into the mitochondria of permanent cell lines and SCO2 deficient patient’s primary cells. Biochim. Biophys. Acta.

[47] Ingegnere T., Mariotti F.R., Pelosi A., Quintarelli C., DE Angelis B., Tumino N., Besi F., Cantoni C., Locatelli F., Vacca P. (2019). Human CAR NK Cells: A New Non-viral Method Allowing High Efficient Transfection and Strong Tumor Cell Killing. Front. Immunol..

[48] Lozzio B.B., Lozzio C.B. (1977). Properties of the K562 cell line derived from a patient with chronic myeloid leukemia. Int. J. Cancer.

[49] Georgiou-Siafis S.K., Samiotaki M.K., Demopoulos V.J., Panayotou G., Tsiftsoglou A.S. (2022). Glutathione-Hemin/Hematin Adduct Formation to Disintegrate Cytotoxic Oxidant Hemin/Hematin in Human K562 Cells and Red Blood Cells’ Hemolysates: Impact of Glutathione on the Hemolytic Disorders and Homeostasis. Antioxidants.

[50] Davies D.M., Foster J., Van Der Stegen S.J.C., Parente-Pereira A.C., Chiapero-Stanke L., Delinassios G.J., Burbridge S.E., Kao V., Liu Z., Bosshard-Carter L. (2012). Flexible Targeting of ErbB Dimers That Drive Tumorigenesis by Using Genetically Engineered T Cells. Mol. Med..

[51] Jemielity J., Fowler T., Zuberek J., Stepinski J., Lewdorowicz M., Niedzwiecka A., Stolarski R., Darzynkiewicz E., Rhoads R.E. (2003). Novel “anti-reverse” cap analogs with superior translational properties. RNA.

[52] Chomczynski P., Sacchi N. (1987). Single-step method of RNA isolation by acid guanidinium thiocyanate-phenol-chloroform extraction. Anal. Biochem..

[53] Muinao T., Pal M., Boruah H.P.D. (2018). Cytosolic and Transmembrane Protein Extraction Methods of Breast and Ovarian Cancer Cells: A Comparative Study. J. Biomol. Tech..

[54] Suski J.M., Lebiedzinska M., Wojtala A., Duszynski J., Giorgi C., Pinton P., Wieckowski M.R. (2014). Isolation of plasma membrane–associated membranes from rat liver. Nat. Protoc..

[55] Laemmli U.K. (1970). Cleavage of structural proteins during the assembly of the head of bacteriophage T4. Nature.

[56] Lai H.-C., Chang C.-J., Yang C.-H., Hsu Y.-J., Chen C.-C., Lin C.-S., Tsai Y.-H., Huang T.-T., Ojcius D.M., Tsai Y.-H. (2012). Activation of NK cell cytotoxicity by the natural compound 2,3-butanediol. J. Leukoc. Biol..

[57] Sarma K.D., Ray D., Antony A. (2000). Improved sensitivity of trypan blue dye exclusion assay with Ni2+ or Co2+ salts. Cytotechnology.

[58] Komatsu F., Kajiwara M. (1998). Relation of natural killer cell line NK-92-mediated cytolysis (NK-92-lysis) with the surface markers of major histocompatibility complex class I antigens, adhesion molecules, and Fas of target cells. Oncol. Res..

[59] Prager I., Watzl C. (2019). Mechanisms of natural killer cell-mediated cellular cytotoxicity. J. Leukoc. Biol..

[60] Ohnishi Y., Minamino Y., Kakudo K., Nozaki M. (2014). Resistance of oral squamous cell carcinoma cells to cetuximab is associated with EGFR insensitivity and enhanced stem cell-like potency. Oncol. Rep..

[61] Melenhorst J.J., Chen G.M., Wang M., Porter D.L., Chen C., Collins M.A., Gao P., Bandyopadhyay S., Sun H., Zhao Z. (2022). Decade-long leukaemia remissions with persistence of CD4+ CAR T cells. Nature.

[62] Pan K., Farrukh H., Chittepu V.C.S.R., Xu H., Pan C.-X., Zhu Z. (2022). CAR race to cancer immunotherapy: From CAR T, CAR NK to CAR macrophage therapy. J. Exp. Clin. Cancer Res..

[63] Whittington M.D., McQueen R.B., Campbell J.D. (2020). Valuing Chimeric Antigen Receptor T-Cell Therapy: Current Evidence, Uncertainties, and Payment Implications. J. Clin. Oncol..

[64] Patel U., Ms J.A., Savani B.N., Oluwole O., Sengsayadeth S., Dholaria B. (2021). CAR T cell therapy in solid tumors: A review of current clinical trials. eJHaem.

[65] Larcombe-Young D., Papa S., Maher J. (2020). PanErbB-targeted CAR T-cell immunotherapy of head and neck cancer. Expert Opin. Biol. Ther..

[66] Futaki S., Nakase I. (2017). Cell-Surface Interactions on Arginine-Rich Cell-Penetrating Peptides Allow for Multiplex Modes of Internalization. Accounts Chem. Res..

[67] Bolhassani A., Jafarzade B.S., Mardani G. (2017). In vitro and in vivo delivery of therapeutic proteins using cell penetrating peptides. Peptides.

[68] Hu J.W., Liu B.R., Wu C.-Y., Lu S.-W., Lee H.-J. (2009). Protein transport in human cells mediated by covalently and noncovalently conjugated arginine-rich intracellular delivery peptides. Peptides.

[69] Papadopoulou L.C., Tsiftsoglou A.S. (2013). The potential role of cell penetrating peptides in the intracellular delivery of proteins for therapy of erythroid related disorders. Pharmaceuticals.

[70] Orlandini von Niessen A.G., Poleganov M.A., Rechner C., Plaschke A., Kranz L.M., Fesser S., Diken M., Löwer M., Vallazza B., Beissert T. (2019). Improving mRNA-Based Therapeutic Gene Delivery by Expression-Augmenting 3’ UTRs Identified by Cellular Library Screening. Mol. Ther..

[71] Fritz S.E., Haque N., Hogg J.R. (2018). Highly efficient in vitro translation of authentic affinity-purified messenger ribonucleoprotein complexes. RNA.

[72] Yoon S.R., Kim T.-D., Choi I. (2015). Understanding of molecular mechanisms in natural killer cell therapy. Exp. Mol. Med..

[73] Meng Y., Yang P., Ma L. (2020). Prognostic and clinical implications of c-erbB-2 expression in patients with oral cancer: A meta-analysis. Medicine.

[74] Yu S., Liu Q., Han X., Qin S., Zhao W., Li A., Wu K. (2017). Development and clinical application of anti-HER2 monoclonal and bispecific antibodies for cancer treatment. Exp. Hematol. Oncol..

[75] Korpela S.P., Hinz T.K., Oweida A., Kim J., Calhoun J., Ferris R., Nemenoff R.A., Karam S.D., Clambey E.T., Heasley L.E. (2021). Role of epidermal growth factor receptor inhibitor-induced interferon pathway signaling in the head and neck squamous cell carcinoma therapeutic response. J. Transl. Med..

[76] Yarden Y., Pines G. (2012). The ERBB network: At last, cancer therapy meets systems biology. Nat. Cancer.

[77] Nozaki M., Yasui H., Ohnishi Y. (2019). Ligand-Independent EGFR Activation by Anchorage-Stimulated Src Promotes Cancer Cell Proliferation and Cetuximab Resistance via ErbB3 Phosphorylation. Cancers.

[78] Ohnishi Y., Yasui H., Nozaki M., Nakajima M. (2018). Molecularly-targeted therapy for the oral cancer stem cells. Jpn. Dent. Sci. Rev..

[79] Zamora A.E., Grossenbacher S.K., Aguilar E.G., Murphy W.J. (2015). Models to Study NK Cell Biology and Possible Clinical Application. Curr. Protoc. Immunol..

[80] Masuda H., Alev C., Akimaru H., Ito R., Shizuno T., Kobori M., Horii M., Ishihara T., Isobe K., Isozaki M. (2011). Methodological Development of a Clonogenic Assay to Determine Endothelial Progenitor Cell Potential. Circ. Res..

