# Supplementary material for: An Innovative PTD-IVT-mRNA Delivery Platform for CAR Immunotherapy of ErbB(+) Solid Tumor Neoplastic Cells
Source: Biomedicines. 2022 Nov 10;10(11):2885. doi: 10.3390/biomedicines10112885 (PMC9687928; doi:10.3390/biomedicines10112885)
Supplement: Supplementary file 1 [file biomedicines-10-02885-s001.zip › biomedicines-2003689-supplementary.pdf]

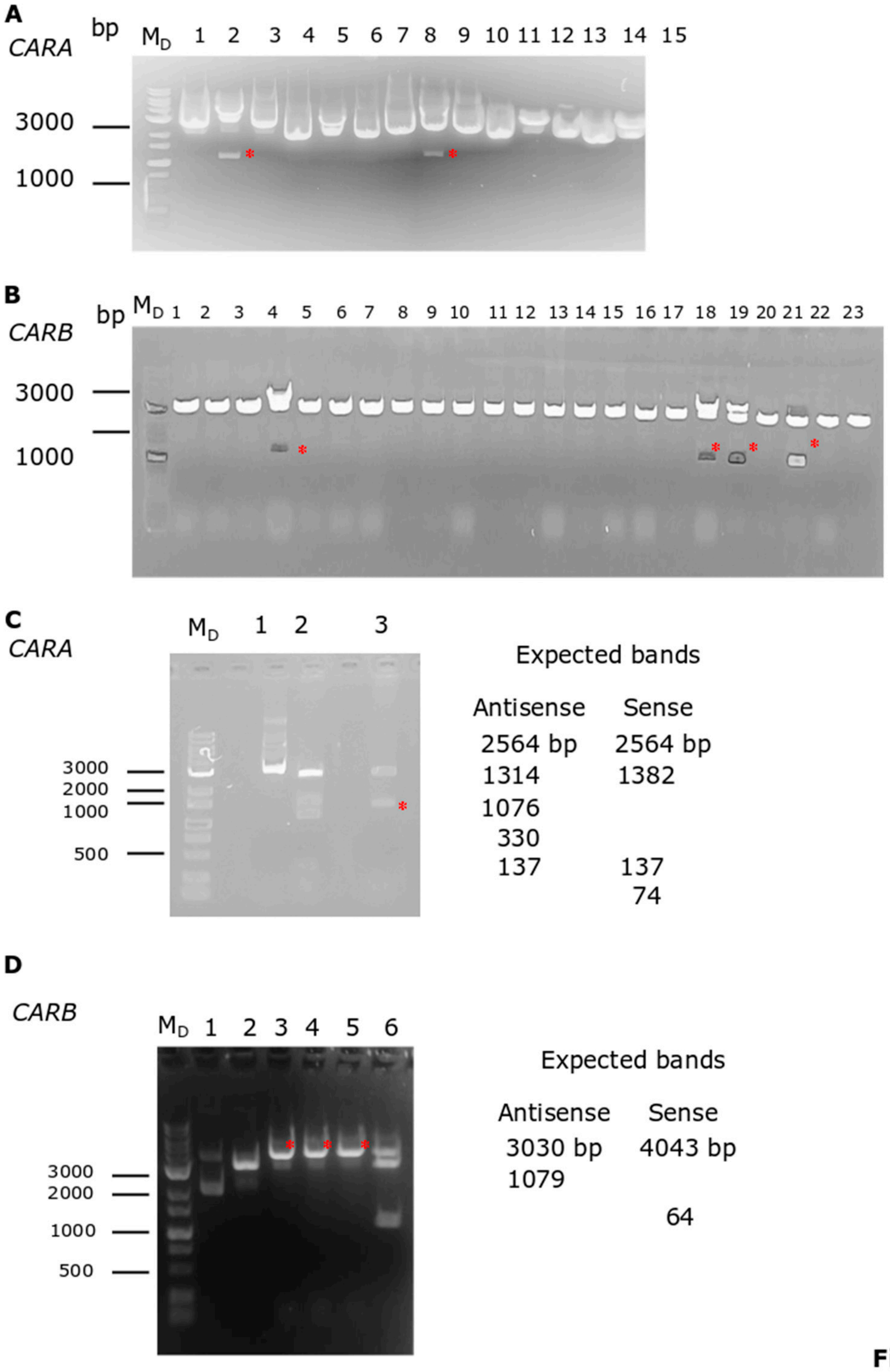

Fig. S1

Figure S1. Assessment of cloning of *CARA* and *CARB* inserts into pGEM vector via proper, sense orientation for in vitro transcription. Subsequent to TA cloning of the inserts corresponding to *CARA* and *CARB* (both inserts at 1130 bp) into pGEM T-easy vector (3105 bp), different colonies were selected to isolate plasmids. (**A-B**) Several clones (1-15, *CARA*), (1-23, *CARB*) were digested with EcoRI and the results of the electrophoresis are shown. EcoRI recognizes at both ends of each insert and the specified clones bearing the insert are labeled. (**C-D**) Two clones of pGEM-*CARA* and four clones for pGEM-*CARB* were digested with either NcoI and PvuII or NcoI, respectively. These digestions were selected to yield different fragments whether the insert was cloned via sense or

antisense orientation. (**C**) Lanes, 1: uncut plasmid; 2–3: different clones of pGEM-*CARA* construct digested with NcoI and PvuII. (**D**) Lanes, 1: uncut plasmid; 2–6: different clones of pGEM-*CARB* digested with NcoI. Clones with sense orientation of inserts were selected for subsequent in vitro transcription. MD: DNA molecular weight marker.

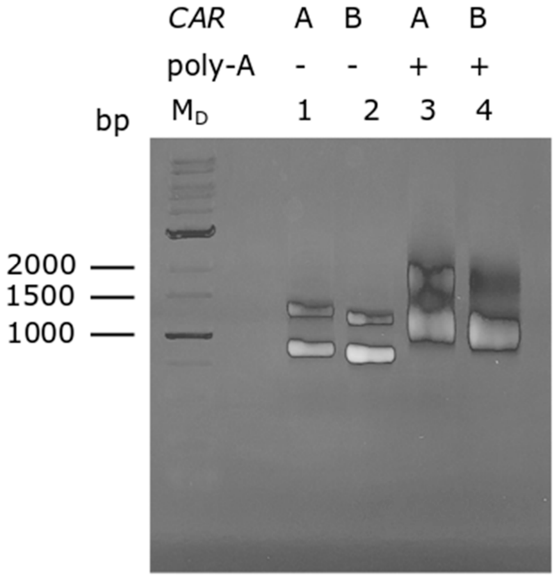

Figure S2. Electrophoresis mobility pattern of *CARA* and *CARB* IVT-mRNAs. At the end of the in vitro transcription, a sample of each IVT mRNA was poly-adenylated. Both samples, prior to and after poly-adenylation, were heat-denatured at 70 °C for 10 min and analyzed in 1% agarose gel. Lanes, MD: DNA molecular weight marker, 1–2: IVT-mRNAs corresponding to *CARA* and *CARB*, 3-4: poly-adenylated IVT-mRNAs for *CARA* and *CARB*, respectively.

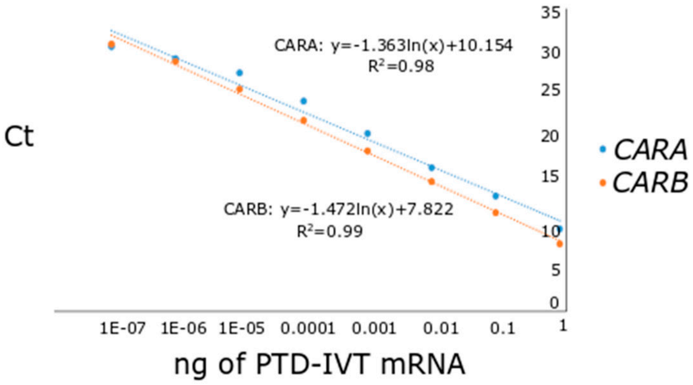

Figure S3. Standard Curves of PTD-IVT-mRNAs for *CARA* and *CARB*: 500 ng of each PTD-IVT-mRNA was phenol-chloroform extracted, precipitated, and reconstituted in water at 12.5 ng/μL. Thereafter 5 μL of the resulting PTD-IVT-mRNA served as the template for cDNA synthesis. Serial dilutions of cDNAs (1/10 to 1/10^7) were amplified by qPCR, employing the corresponding primers. Each assay was performed in duplicate. Standard curve created and intracellular quantity of PTD-IVT-mRNAs was calculated (as shown at Figure 5).

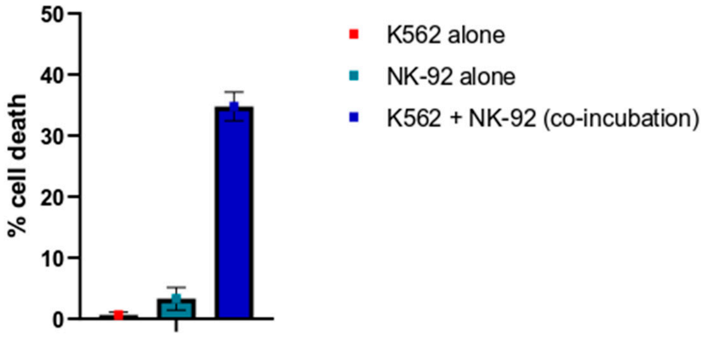

Figure S4. Assessment of NK-92-cell-induced cytotoxicity on K562 cells. K562 cells (1 × 10^5 cells) were co-incubated with NK-92 cells (1 X10^6 cells) in the cell culture medium of NK-92 cells for 6 h. K562 and NK-92 cells cultured also separately as controls. Then, samples of each culture were removed, and cell viability was assessed by the trypan blue exclusion assay.

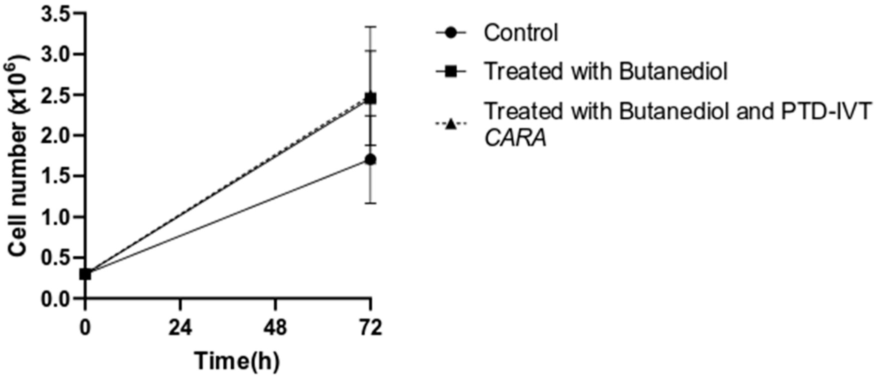

Figure S5. Cell growth of NK-92 cells treated with 2,3-butanediol in the presence and absence of PTD-IVT-mRNA of *CARA*. NK-92 cells seeded at 0.3 × 10^6 (time 0 h) were treated with 2,3-butanediol (at 10 μM) in presence and absence of PTD-IVT-mRNA of *CARA*. Cell growth was assessed at 72h by flow cytometry. Cell growth in a culture of NK-92 cells left untreated was assessed in parallel.

A

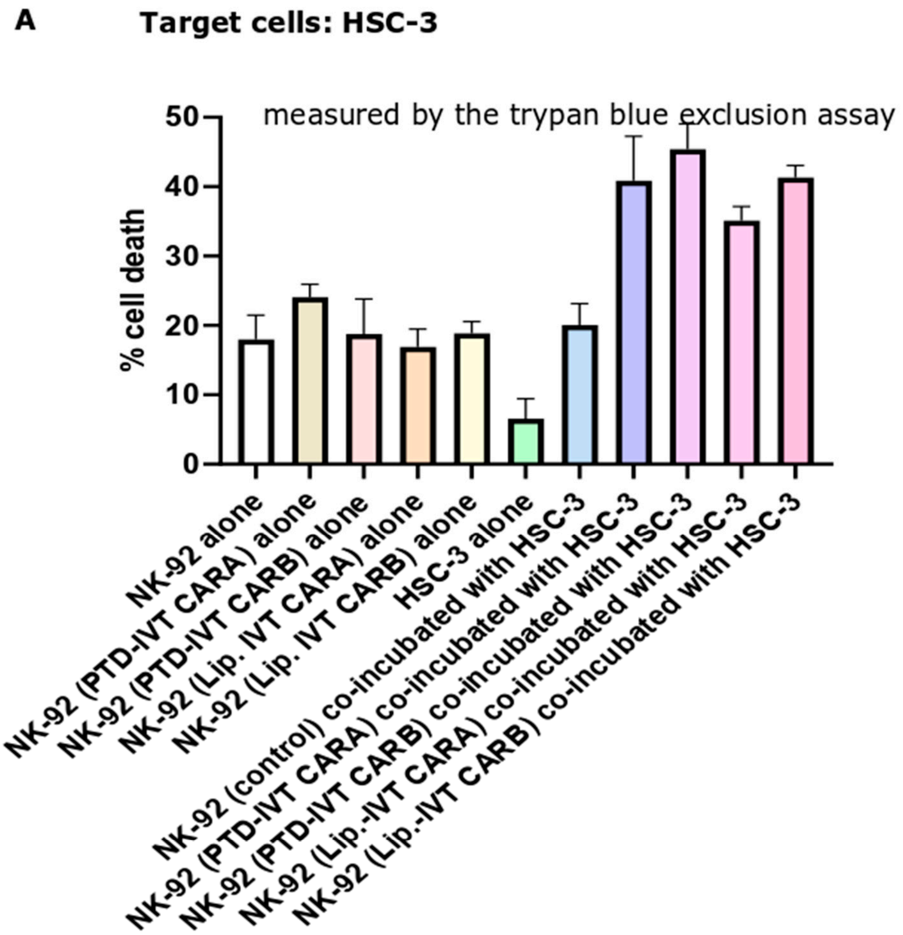

B

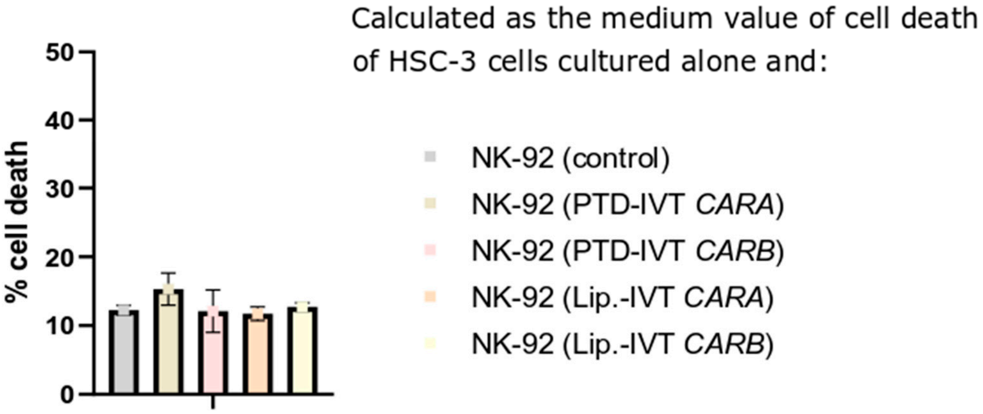

Figure S6. Cell death percentage of the different experimental treatments of HSC-3 and/or NK-92 cells (engineered or not) cultured separately or co-incubated simultaneously. (A) Percentages of cell death as measured by the trypan blue exclusion assay at the different treatments are presented. These data served as the raw data for the calculation of the results of Figure 7A. (B) Percentage of cell death calculated by cultures of HSC-3 cells as well as NK-92 cells (different treatments), all cultured separately, as described in Figure 7A. These data served as percentage of cell death of mixed sub-populations, without the co-incubation period.

A **Target cells: HSC-3**

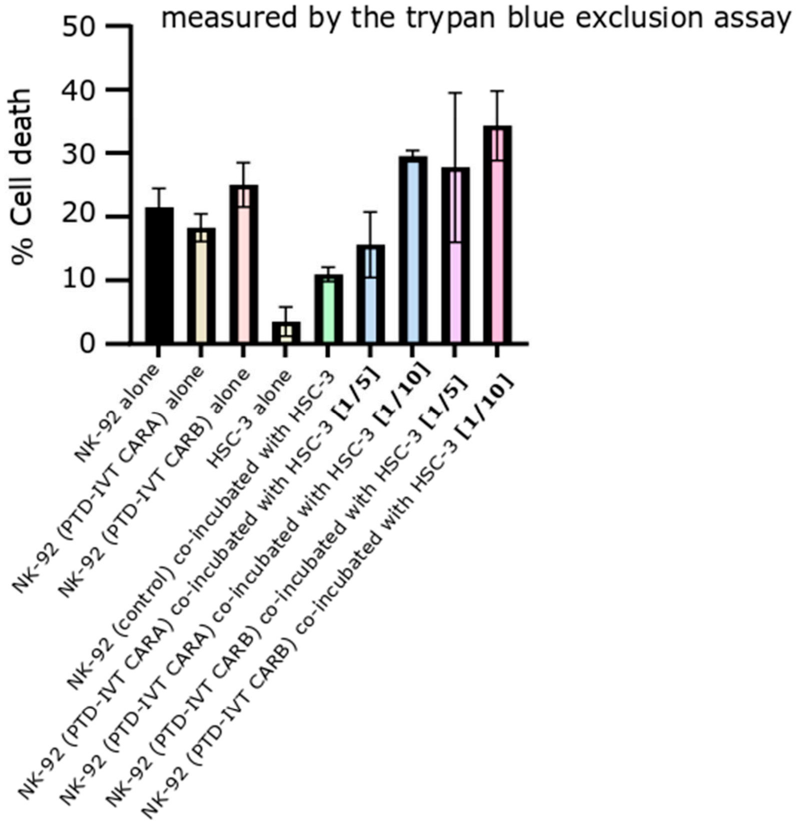

B

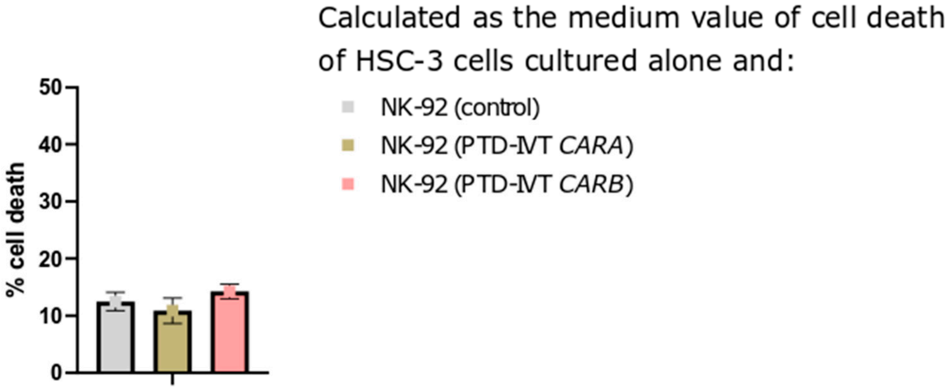

Figure S7. Cell death (raw and calculated results), presented in Figure 7C.

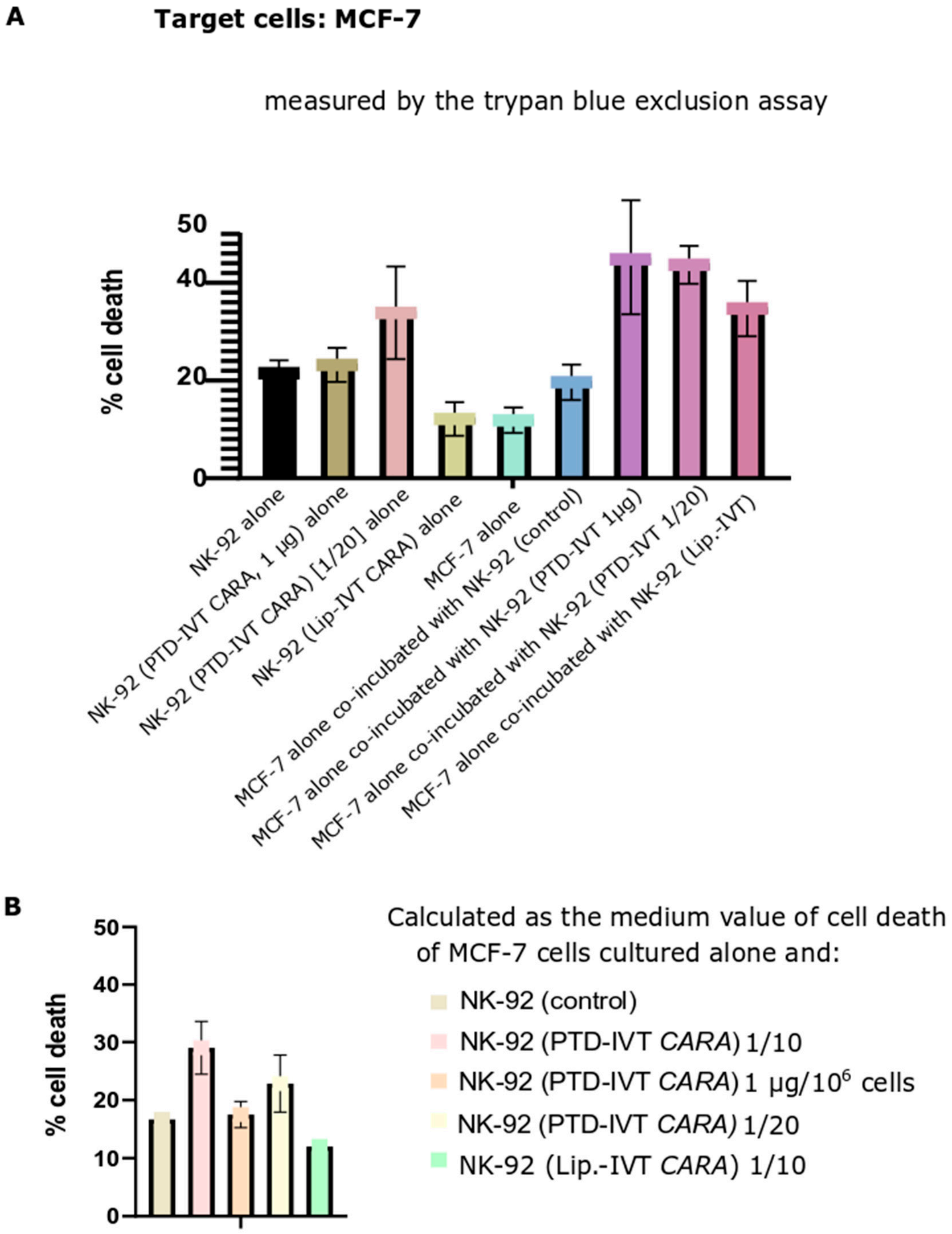

Figure S8. The raw results of the NK-92-induced cytotoxicity on MCF-7 cells, as presented in Figure 9C.
